# Association between Domperidone Administered via Feeding Tube and Feeding Success in Critically Ill Patients with Enteral Feeding Intolerance

**DOI:** 10.3390/jpm11090846

**Published:** 2021-08-27

**Authors:** Yisong Cheng, Chaoyue Chen, Hao Yang, Min Fu, Xi Zhong, Bo Wang, Zhi Hu, Min He, Zhongwei Zhang, Xiaodong Jin, Yan Kang, Qin Wu

**Affiliations:** 1 Department of Critical Care Medicine, West China Hospital, Sichuan University, Chengdu 610064, China; chengyisong2020@wchscu.cn (Y.C.); nonamedoctor@163.com (H.Y.); carrie_613@163.com (M.F.); zhongxivip2006@163.com (X.Z.); wchicu@126.com (B.W.); huzhi1111@hotmail.com (Z.H.); hemin19910306@wchscu.cn (M.H.); zhangzhongwei@scu.edu.cn (Z.Z.); zh_jxd@163.com (X.J.); kangyan@scu.edu.cn (Y.K.); 2 Department of Neurosurgery, West China Hospital, Sichuan University, Chengdu 610064, China; chaoyuechen01@scu.edu.cn

**Keywords:** enteral nutrition, domperidone, metoclopramide, feeding success, efficacy, safety

## Abstract

One nutritional challenge in critically ill patients is enteral feeding intolerance (EFI), but current prokinetic agents have uncertain efficacy and safety profiles. We conducted a longitudinal, single-center, retrospective study to evaluate the efficacy and safety of domperidone administered via the feeding tube versus intravenous (IV) metoclopramide among adult patients with EFI. The primary outcome was feeding success, defined as the proportion of patients with average percentage of daily protein prescription >80% of the target dose. The secondary outcomes were safety endpoints. Among 28,814 intensive care unit (ICU) admissions, 552 patients with EFI were included, 38 receiving IV metoclopramide and 514 receiving tube feeding domperidone. The proportion of feeding success in patients receiving tube feeding domperidone and IV metoclopramide was 42.02% and 21.05%, respectively. After 1:2 matching (IV metoclopramide to tube feeding domperidone), the proportion of feeding success was 40.79% in patients receiving tube feeding domperidone. Basically, after matching, there were no differences in any safety endpoints (mortality and length of stay during ICU and hospitalization, organ-support-treatment free days) or adverse events (recurrence of EFI, electrolyte disturbance, abdominal and other symptoms) between the two groups (*p* > 0.05). A logistic regression analysis in the matched cohort indicated that domperidone administered via the feeding tube was independently associated with feeding success. We found that tube feeding domperidone was efficient in increasing enteral nutrition delivery performance among critically ill adult patients with EFI.

## 1. Introduction

Intensive care unit (ICU) patients often exhibit metabolic changes arising through critical illness that result in accelerated catabolism, and consequently enteral nutrition is a consideration for all ICU patients [1]. Although the optimal daily amounts of energy and protein remain uncertain, nutrition delivery by enteral feeding remains suboptimal as a result of interruptions for various reasons. This may impact the provision of calories and proteins and, therefore, the clinical outcome [2]. A challenge in enteral nutrition feeding practices is enteral feeding intolerance (EFI), defined as the inability to provide adequate enteral nutrition to critically ill patients due to delayed gastric emptying without mechanical obstruction. Due to the lack of a universal definition, the incidence of EFI in adult ICU patients has been reported at 24% to 30%, in various ICU populations [3,4].

In critically ill patients with EFI, it is widely accepted that intravenous (IV) erythromycin, IV metoclopramide, or their combination can be used as prokinetic therapy to reduce the gastric residual volume, thereby improving feeding performance [1,5]. The clinical use of erythromycin is limited by antibiotic regulation policy, QT prolongation, and super-infection with multi drug-resistant organisms [6]; metoclopramide represents the standard of care for EFI treatment at most institutions [7]. However, metoclopramide causes blockade of dopamine D2 receptors in the central nervous system, which can produce a variety of side effects [8]. Oral domperidone is a peripherally selective D2 receptor antagonist that is widely used in east Asian countries to help with stomach emptying in people with delayed gastric emptying [3,9]. Unlike other D2 receptor antagonists, domperidone exhibits minimal crossing of the blood-brain-barrier and is less likely to cause side effects [10,11]. Studies have shown that domperidone administered via the feeding tube and IV metoclopramide are effective in improving the success rate of post-pyloric feeding tube placement in critically ill patients [12,13]. However, the safety and efficacy of domperidone as a prokinetic drug administered to EFI patients via a feeding tube remains uncertain. The purpose of this trial was to compare the efficacy of domperidone administered via the feeding tube and IV metoclopramide in critically ill patients with EFI.

## 2. Results

### 2.1. Patient Demographic Characteristics

**Figure 1:**
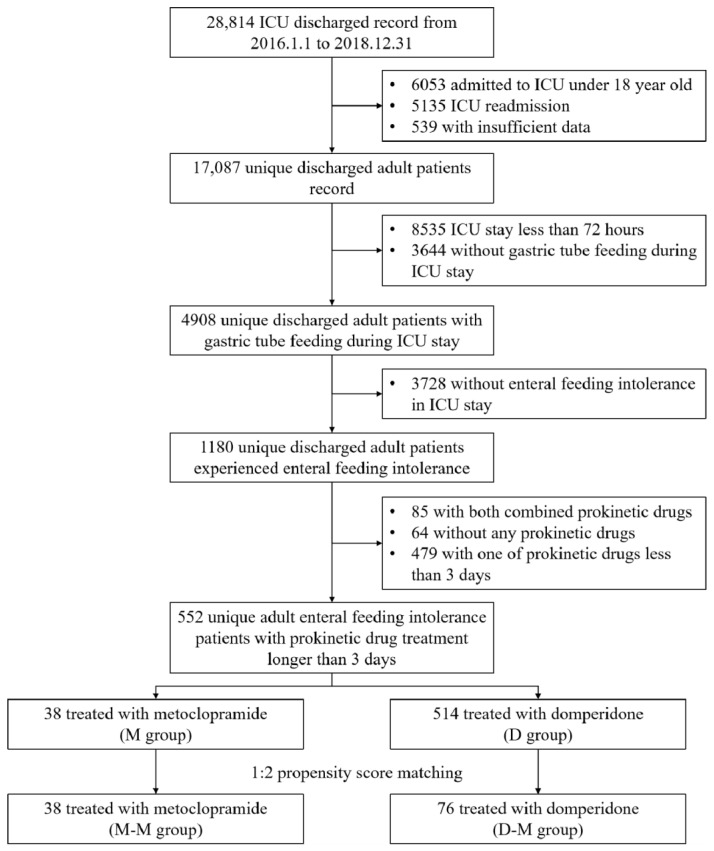
Study profile.

A flowchart for the study is shown in Figure 1. After reviewing 28,814 records for first ICU admissions among patients aged ≥18 years, we identified 552 EFI patients who received either one of the two prokinetic drugs: 38 received IV metoclopramide (M group) and 514 received domperidone via the feeding tube (D group).

The characteristics of the cohort are summarized in Table 1. Patients in D group had significantly higher Sequential Organ Failure Assessment score (SOFA), shorter hospitalization time before ICU admission, earlier occurrence of EFI after initiation of tube feeding, and higher rate of opioid exposure (23.15% vs. 7.89%, *p* = 0.029) when starting prokinetic treatment. Additional baseline data are presented in the supplemental files (Tables S1–S3). During the observation period (shown in Table S4), more patients in the D group received fentanyl (39.2% vs. 13.16%, *p* = 0.002) and propofol (47.47% vs. 28.95%, *p* = 0.041) while more patients in the M group received Kabiven (injectable amino acid, electrolyte, dextrose, and lipid emulsion for IV use, Fresenius Kabi USA, Lake Zurich, IL, USA) (36.84% vs. 16.54%, *p* = 0.003).

APACHE II: The Acute Physiology and Chronic Health Evaluation II. BMI: Body mass index. CRRT: Continuous Renal Replacement Therapies. D group: domperidone group. D-M group: domperidone group after propensity matching. M group: metoclopramide group. M-M group: metoclopramide group after propensity matching. NUTRIC: The Nutrition Risk in Critically ill score. SOFA: The Sequential Organ Failure Assessment score. SD: standard deviation.

### 2.2. Efficacy in the Unmatched Cohort

**Figure 2:**
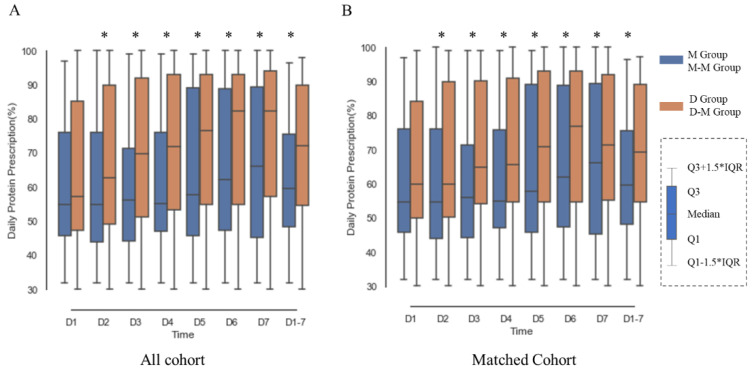
Percentage of daily protein prescription in protein goal (DPP%) through the observational period in (**A**) unmatched cohort and (**B**) 1:2 propensity score matching cohort based on Model 1. (**A**) In the unmatched cohort, the mean DPP% was significantly higher (marked with an asterisk) in the tube-feeding domperidone group compared with IV metoclopramide group on day 2 (67.72 vs. 59.71, *p* = 0.024), day 3 (70.93 vs. 60.15, *p* = 0.002), day 4 (71.74 vs. 61.65, *p* = 0.005), day 5 (73.41 vs. 64.91, *p* = 0.026), day 6 (74.75 vs. 65.61, *p* = 0.009), and day 7 (75.61 vs. 65.20, *p* = 0.006), as well as average DPP% (71.11 vs. 62.43, *p* = 0.007). (**B**) In matched cohort, the mean DPP% was significantly higher (marked with an asterisk) in the tube-feeding domperidone group compared with IV metoclopramide group on day 2 (68.70 vs. 59.71, *p* = 0.019), day 3 (71.06 vs. 60.15, *p* = 0.004), day 4 (71.96 vs. 61.65, *p* = 0.005), day 5 (74.53 vs. 64.91, *p* = 0.009), day 6 (76.10 vs. 65.61, *p* = 0.008), and day 7 (76.49 vs. 65.20, *p* = 0.002), as well as average DPP% (71.77 vs. 62.43, *p* = 0.006). IQR: interquartile range. *: *p* < 0.05.

As the primary outcome, the feeding success rate in the unmatched cohort was 216 (42.02%) in the D group and 8 (21.05%) in the M group (Table 2). In detail, patients in the D group had a significantly higher percentage of protein delivery relative to the total protein goal from day 2 than patients who received IV metoclopramide (Figure 2A). The average DPP% differed significantly between the two groups (metoclopramide 62.42% vs. domperidone 71.11%, *p* = 0.007, Figure 2A). The proportion of patients who achieved 80% of the target calories was significantly higher in the D group compared with the M Group from days 2 to 4 (Figure S1C). The volume of enteral nutrition was also higher in the D group compared with the M group for most of the observation period, as were the absolute amount of protein and calories delivered (Figure S2).

### 2.3. Safety in the Unmatched Cohort

The data for the safety endpoints are shown in Table 2; there was no statistically significant difference in recurrence of EFI, ICU mortality, ICU LOS and cost, ventilation/vasopressor/continuous renal replacement therapy (CRRT)-free days, proportions of adverse events (new onset atrial fibrillation, diarrhea, constipation), and laboratory examination between group D and M. However, we found that the elevated creatine kinase rate was higher in group D compared to group M (51.17% vs. 26.32, *p* < 0.001).

### 2.4. Predictors of Feeding Success in the Unmatched Cohort

To determine factors associated with average DPP% > 80% of the target, univariate and multivariate backward logistic regression analyses were performed (Table S5 and Table 3). Domperidone (OR = 3.001, 95% CI: 1.334–6.755, *p* = 0.008), exposure to opioids (OR = 1.723, 95% CI: 1.132–2.263, *p* = 0.011), and placement of nasoenteric tube (OR = 0.545, 95% CI: 0.334–0.892, *p* = 0.023) were associated with feeding success.

### 2.5. Propensity Score-Matched Cohort

The propensity scores matched the cohort from the primary analysis (Table 1), comprising 114 patients: 38 in the IV metoclopramide group and 76 in the tube feeding domperidone group. Covariate differences between the groups were compared after matching (Table 1 and Tables S2–S4). The results demonstrated that more patients in the matched domperidone group were: exposed to opioid treatment before starting prokinetic drugs (30.26% vs. 7.89%, *p* = 0.007), received treatment with fentanyl during the observation period (34.21% vs. 13.16%, *p* = 0.031), had higher average daily doses of propofol (198.67 ± 391.09 vs. 45.75 ± 120.25, *p* = 0.020), had less malignancy (2.64% vs. 15.79%, *p* = 0.028), and received less Kabiven (15.79% vs. 36.84%, *p* = 0.022).

### 2.6. Primary and Secondary Outcomes in the Matched Cohort

In the overall propensity score-matched cohort, 31 (40.79%) patients in the D-M group and 8 (21.05%) patients in the M-M group reached average DPP% > 80% of the target goal during the observation period (Table 2). The percentages of protein delivery relative to the target protein goal are shown in Figure 2B.

In the matched cohort, the safety endpoints analogously showed that EFI recurrence, ICU mortality, ICU LOS and costs, proportions of adverse events, and laboratory examinations findings were similar between the D-M and M-M group (*p* > 0.05).

### 2.7. Predictors of Feeding Success and Sensitivity Analysis in the Matched Cohort

Logistic regression analysis in the matched cohort indicated that domperidone administered via the feeding tube was independently associated with feeding success (OR = 2.745, 95% CI: 1.094–6.888, *p* = 0.031, Table 4). To address the concern that any significant differences may be ascribed to the 1:2 propensity score matching, different matching ratios between the patient characteristics were performed for sensitivity analyses. Patients receiving domperidone via the feeding tube still had a significantly higher proportion of average DPP% > 80% of the target as well as percentage of protein delivery relative to the protein goal (Table S6 and Figure S3). In another propensity score-matched cohort based on statistical model 2 (see Supplementary Materials), the primary outcome remained significantly different in the D group (Table S7 and Figure S4).

## 3. Discussion

To the best of our knowledge, this retrospective study is the first study to compare efficacy and safety between IV metoclopramide and domperidone administered via the feeding tube in critically ill patients with EFI. We found that tube feeding domperidone had a higher feeding success rate than IV metoclopramide, however, no differences were noted in secondary safety endpoints. Among critically ill adult patients with EFI, domperidone seems to be efficient in increasing enteral nutrition delivery performance and does not increase the risk of adverse events.

Prokinetic drugs are important in the treatment of functional gastrointestinal disorders and are used off-label in critically ill patients to improve gastric emptying [6]. Although metoclopramide and erythromycin have become the standard treatments for patients with EFI in most ICUs, their side effects limit their clinical use, and thus new agents are still needed to improve feeding performance in patients at high risk for aspiration and critical illness-associated gastric motility dysfunction [14].

Compared with IV metoclopramide, we observed that domperidone administered via the feeding tube increased the proportion of patients who met 80% of the target protein goal by increasing the volume of nutrition delivery without increasing the risk of adverse events. Average DPP% > 80% of the target goal was chosen as the primary outcome because protein was recently identified as one of the most important nutritional factors impacting ICU outcomes [15,16]. In the present study, there was no statistical difference in the recurrence rate of EFI in the unmatched cohort and matched cohort. Considering the confounding factors within the retrospective study design, we used a propensity score-matching method based on different ratios and variables to test the sensitivity of our conclusion. The primary efficacy outcome remained significant in all models tested, and the safety endpoints for tube-feeding domperidone and IV metoclopramide were comparable.

Previous studies have compared the efficacies of metoclopramide and domperidone in other diseases impacting gastric motility. Similar to the present study, the effect of domperidone was suggested to be superior to that of metoclopramide for symptoms of diabetic gastroparesis, pediatric vomiting, and others [17,18]. One study demonstrated a dose–response relationship for the effect of domperidone [19]. A recently published parallel-group trial on patients with septic shock showed no significant difference in terms of gastric electrical rhythm measured by surface electrogastrography [20]. Taken together, the present study indicates the potential for domperidone administered via the feeding tube in the treatment of patients with EFI, but the optimal dose, route of administration, and comparable dose of metoclopramide should be taken into consideration when designing future studies.

In the current study, tube feeding domperidone was used as the first line prokinetic therapy while IV metoclopramide was used in patients with a high risk of vomiting due to cancers and other reasons, and the physician determined that domperidone is not suitable for these patients. Thus, the proportion of malignancy in the M-M group was still higher than the D-M group after propensity score matching. However, the remaining clinical characteristics and feeding conditions were all matched.

The safety of domperidone needs to be monitored. Domperidone has had its status withdrawn in some countries because of potentially life-threatening cardiac effects in patients aged above 60 years, but it is still commonly used in some Asian countries [21]. In the present study, we did not observe increased overall mortality in the domperidone group, and subgroup analyses demonstrated no significant difference in mortality for patients aged above 60 years (Table 2). It remains unclear whether domperidone exposure definitely increases the risk of sudden cardiac death and death associated with ventricular arrhythmia [22]. Some studies have indicated a risk for severe cardiac adverse effects after exposure to both metoclopramide and domperidone [22,23]. It has been suggested that domperidone may not be associated with an increased risk of cardiovascular events at doses below 30 mg/day and does not result in QT prolongation [24]. In other functional gastrointestinal disorders, both domperidone and metoclopramide may induce central nervous system (CNS) side effects and symptoms [25]. In the present study, the incidence of delirium was similar in the two groups, and further studies are needed to explore the potential associations between prokinetic agents and cardiovascular adverse events and CNS side effects.

A recent study compared a ghrelin agonist and metoclopramide in critically ill patients with EFI and found no differences in feeding outcomes or adverse events [26]. Compared with that study, the patients in the present study achieved lower rates of feeding success; however, this is partly due to patients’ withdrawals in that study, resulting in a reduction in the number of patients remaining in the trial. Another study indicated that enteral nutrient delivery was suboptimal in Chinese ICUs because of a lack of or differences in standardized feeding protocols, which may make it difficult to compare the present data with other studies [27]. However, we adopted a volume-based feeding strategy, with reduced energy and increased protein density, which is popular in our hospital, perhaps because of a fear of hyperglycemia. A prospective study based on a more general feeding approach is needed to verify our findings.

The present study raises some additional questions. Metoclopramide and domperidone theoretically share similar pharmacological action, but the detailed mechanism is unclear. Studies have indicated that combination therapy with metoclopramide and neostigmine can decrease the gastric resident volume in critically ill patients with greater efficacy than each monotherapy, which raises the question of whether it is more efficient to combine domperidone and erythromycin than to use with metoclopramide or erythromycin alone, as recommended by the current guideline [28]. In the present study, we only included patients who used either prokinetic drug for more than 3 days. One of the problems with prokinetics agents is the occurrence of tachyphylaxis, which further raises the question of whether short-term administration (e.g., less than 3 days) would be better than long-term administration.

There are several weaknesses to the present study. First, it was a retrospective study. Therefore, even though the patients’ data were collected from a large database, the patient selection may still have had some bias. Second, we could not fully investigate the side effects of the two drugs, especially QT prolongation interval and some outcomes including vomiting because of unavailable data. Third, the definition used for EFI in this study was a combined definition (GRV ≥ 500 mL), which did not fully conform to the definitions in other studies. Some patients with GRV less than 500 mL may also have EFI due to gastric distention, abdominal cramping, regurgitation/emesis, etc, thus, these patients may have been overlooked. Fourth, although all of the patients were enrolled from the same center and fed by standard feeding protocol, the feeding success rate and the protein delivered were lower than other studies, which may impede wider extrapolation of our conclusions. Fifth, the relatively small size of the IV metoclopramide group may have affected the reliability of the results. Finally, the limited length of follow-up in the patients may have prevented more conclusive results in this patient population.

## 4. Methods

### 4.1. Study Population and Design

This study is reported in accordance with the Strengthening the Reporting of Observational studies in Epidemiology (STROBE) statement and was designed to investigate whether domperidone administered via the feeding tube contributes to improvements in enteral nutrition feeding performance compared with IV metoclopramide in patients with EFI [29]. We conducted a longitudinal, single-center, retrospective study on adult patients in the Department of Critical Care Medicine, West China Hospital. The study was approved by the Institutional Review Board at West China Hospital and was granted a waiver of informed consent (2019-S-361).

The study cohort was derived from all unique patients discharged from the ICU between January 2016 and December 2018. We included patients who experienced at least one episode of EFI and received either IV metoclopramide or domperidone administered via the feeding tube to improve feeding performance after the occurrence of EFI. All patients in our department received a standard feeding protocol. Patients with EFI either received domperidone or metoclopramide; in our center, domperidone administered via the feeding tube was the first line prokinetic therapy and IV metoclopramide was selected if the patient was not suitable for tube-fed domperidone judged by a physician. For example, for a cancer patient with EFI receiving chemotherapy and/or radiotherapy, the physician evaluated that the patient had a high risk of vomiting and would choose IV metoclopramide then. EFI was defined as gastric residual volume ≥ 500 mL on one measurement. The exclusion criteria were: (1) missing data; (2) age < 18 years; (3) ICU stay < 3 days; (4) more than one type of prokinetic drug during the observation period; (5) prokinetic drug treatment < 3 days; and (6) exposure to any prokinetic medications within 48 h before EFI occurrence. The observation period was defined as 7 days after prokinetic drug administration started. For patients who had more than one ICU stay, the first ICU admission record was kept. For patients who experienced repeated EFI during the ICU stay, only records for the first episode were analyzed and considered as EFI recurrence. SOFA and the Acute Physiology and Chronic Health Evaluation II (APACHE II), with higher scores indicating more severe disease and a higher risk of death, were evaluated by the attending physician who saw the patients as far as being transferred to ICU. The Nutrition Risk in Critically ill score (NUTRIC:) were calculated by the highest recording values of each variable during the 24 h after ICU admission as a reference for nutritional therapy.

### 4.2. Feeding Policy and Prokinetic Drug Therapy

A volume-based feeding protocol was employed in our department. The protein and calorie targets for each patient in the present study were 1.3 g/kg and 25 kcal/kg estimated dry body weight per day, respectively (details provided in the Supplementary Materials).

Patients with EFI received either domperidone (10 mg administered via tube feeding every 8 h) or metoclopramide (10 mg administered as a 50-mL IV infusion over 30 min every 8 h) [11]. The dose of IV metoclopramide was 50% of normal dose in patients with creatinine clearance ≤40 mL/min and 25% in patients with clearance ≤10 mL/min or undergoing dialysis or continuous renal replacement techniques [30].

### 4.3. Primary and Secondary Outcomes

The primary outcome was feeding success, defined as the proportion of patients whose average percentage of daily protein prescription (average DPP%) was >80% of the target dose. The secondary outcomes were safety endpoints, and included: ICU and hospital length of stay; hospital costs; ICU mortality; number of mechanical ventilation-, vasopressor- and continuous renal replacement therapy-free days within 28 days of ICU admission; EFI recurrence; new onset atrial fibrillation, diarrhea, constipation, hyperglycemia, elevated creatine kinase, elevated cardiac troponin T, hyper-/hypokalemia, hyper-/hypomagnesemia, hyper-/hypophosphatemia, or delirium during the observation period; daily calories, protein, and enteral nutrient volume; and proportion of patients who met the 80% goal for protein and calories. Detailed calculations and definitions of outcomes are listed in the Supplementary Materials.

### 4.4. Propensity Score Matching and Grouping

Because of the unbalanced proportions of patients receiving tube feeding domperidone (D group) and IV metoclopramide (M group) in the present study, we performed propensity score-matching to better control for confounding variables. Patients who received tube feeding domperidone and IV metoclopramide were first matched in a 1:2 ratio based on the factors associated with primary outcomes as well as factors of clinical interest (details provided in the Supplementary Materials). Briefly, potential confounders in the matched cohort group were selected based on logistic regression and clinical interests. In Model 1, we matched patients based on baseline factors (age, gender, comorbidity etc.) and laboratory indicators (platelet, total bilirubin, C-reaction protein etc.) before prokinetic treatment with *p* < 0.10. In Model 2, we matched patients based on baseline factors (age, gender, APACHE II etc.) and laboratory indicators (platelet, total bilirubin, PH, HCO_3_ etc.) at ICU admission with *p* < 0.10. One-to-one nearest neighbor matching was performed between groups without replacement using a caliper width of 0.20 times of the standard deviation of the logit of the propensity score. The unmatched cohort and matched cohorts were named as follows:

M group: metoclopramide group.

D group: domperidone group.

M-M group: metoclopramide group after propensity score matching.

D-M group: domperidone group after propensity score matching.

### 4.5. Statistical Methods

We obtained data on sociodemographic characteristics, disease severity, medical comorbidities, laboratory test findings, and treatment characteristics for use in assessment of potentially influential covariates. Results of laboratory tests with missing values exceeding 10% were excluded. We used unpaired 2-tailed *t* tests, or χ^2^ tests, as appropriate, to test the significance of differences between continuous and categorical variables. Logistic regression analysis was used to assess the association between treatment and primary outcomes. All statistical analyses and figures were generated by Python 3.7.0 (Python Software Foundation, Beaverton, OR, USA), and *p* < 0.05 was considered statistically significant.

## 5. Conclusions

In this retrospective study on critically ill patients with EFI, we found that domperidone administered via the feeding tube was efficient in increasing enteral nutrition delivery performance and did not increase the risk of adverse events.

## Figures and Tables

**Table 1:** Baseline characteristics.

	Unmatched Cohort				Matched Cohort			
	All (*n* = 552)	M Group (*n* = 38)	D Group (*n* = 514)	*p*	All (*n* = 114)	M-M Group (*n* = 38)	D-M Group (*n* = 76)	*p*
Demographics								
Age, years, mean ± SD	54.15 ± 16.69	56.03 ± 17.40	54.01 ± 16.64	0.474	56.65 ± 18.34	56.03 ± 17.40	56.96 ± 18.89	0.799
Female, *n* (%)	183 (33.15)	15 (39.47)	168 (32.68)	0.497	36 (31.58)	15 (39.47)	21 (27.63)	0.285
Male, *n* (%)	369 (66.85)	23 (60.53)	346 (67.32)	0.497	78 (68.42)	23 (60.53)	55 (72.37)	0.285
Body weight, kg, mean ± SD	65.81 ± 13.89	62.9 ± 11.13	66.01 ± 14.05	0.270	66.56 ± 14.33	62.9 ± 11.13	68.88 ± 15.73	0.097
BMI, kg/m^2^, mean ± SD	23.29 ± 2.69	22.93 ± 2.38	23.32 ± 2.71	0.394	23.27 ± 1.82	22.93 ± 2.38	23.44 ± 1.45	0.163
Risk scores after ICU admission, mean ± SD								
APACHE II	18.77 ± 7.38	17.21 ± 6.72	18.88 ± 7.42	0.200	18.56 ± 6.53	17.21 ± 6.72	19.19 ± 6.39	0.144
SOFA	7.43 ± 3.13	6.29 ± 2.70	7.51 ± 3.15	0.020	6.10 ± 2.55	6.29 ± 2.70	6.00 ± 2.48	0.569
NUTRIC	4.29 ± 1.49	4.00 ± 1.66	4.32 ± 1.48	0.210	4.04 ± 1.6	4.0 ± 1.66	4.05 ± 1.58	0.869
Admission type ^‡^, *n* (%)				0.995				0.642
Surgical	269 (48.73)	19 (50.0)	250 (48.64)		52 (45.61)	19 (50.0)	33 (43.42)	
Medical	283 (51.27)	19 (50.0)	264 (51.36)		62 (54.39)	19 (50.0)	43 (56.58)	
Admission reason, *n* (%)				0.335				0.251
Post elective surgery	199 (36.10)	15 (39.47)	184 (35.79)		39 (34.21)	15 (39.47)	24 (31.57)	
Post emergency surgery	48 (8.70)	2 (5.26)	46 (8.94)		9 (7.89)	2 (5.26)	7 (9.21)	
From ward	97 (17.60)	5 (13.16)	92 (17.90)		22 (19.29)	5 (13.16)	17 (22.37)	
From emergency department	194 (35.10)	14 (36.84)	180 (35.02)		41 (35.96)	14 (36.84)	27 (35.53)	
Direct transfer from other hospital	14 (2.50)	2 (5.26)	12 (2.33)		3 (2.63)	2 (5.26)	1 (1.32)	
Time before ICU admission, day, mean ± SD	4.45 ± 5.63	6.53 ± 6.30	4.30 ± 5.55	0.018	5.73 ± 5.66	6.53 ± 6.30	5.33 ± 5.31	0.289
Feeding start after admission, day, mean ± SD	2.2 ± 3.3	2.16 ± 2.10	2.20 ± 3.37	0.942	1.86 ± 2.35	2.16 ± 2.10	1.71 ± 2.46	0.339
Time prokinetics started after admitted to ICU, day, mean ± SD	4.45 ± 5.63	6.53 ± 6.30	4.30 ± 5.55	0.018	5.73 ± 5.82	6.53 ± 6.30	5.33 ± 5.57	0.303
Clinical situation when prokinetics started ^∫^, *n* (%)								
Opioids ^∫∫^	122 (22.10)	3 (7.89)	119 (23.15)	0.029	26 (22.81)	3 (7.89)	23 (30.26)	0.007
Propofol	42 (8.04)	2 (5.26)	40 (7.78)	0.572	7 (6.14)	2 (5.26)	5 (6.57)	0.076
Muscle relaxants	2 (0.38)	0 (0.00)	2 (0.38)	0.700	0 (0.00)	0 (0.00)	0 (0.00)	-
CRRT	15 (2.72)	1 (2.63)	14 (2.72)	0.629	2 (1.75)	1 (2.63)	1 (1.32)	0.801
Vasopressor	39 (7.07)	1 (2.63)	38 (7.39)	0.437	4 (3.51)	1 (2.63)	3 (3.95)	0.857
ICU admission diagnosis, *n* (%)								
Sepsis	34 (6.16)	4 (10.53)	30 (5.84)	0.418	11 (9.65)	4 (10.53)	7 (9.21)	0.911
Multi-trauma	10 (1.81)	1 (2.63)	9 (1.75)	0.812	3 (2.63)	1 (2.63)	2 (2.63)	1.000
Brain hemorrhage ^†^	108 (19.57)	11 (28.95)	97 (18.87)	0.194	35 (30.70)	11 (28.95)	24 (31.58)	0.943
Severe acute pancreatitis	38 (6.88)	4 (10.53)	34 (6.61)	0.557	7 (6.14)	4 (10.53)	3 (3.95)	0.334
Pulmonary infection	388 (70.29)	27 (71.05)	361 (70.23)	0.938	88 (77.19)	27 (71.05)	61 (80.26)	0.385
Comorbidity, *n* (%)								
Malignancy	71 (12.86)	6 (15.79)	65 (12.65)	0.758	8 (7.02)	6 (15.79)	2 (2.63)	0.028
Hypertension	98 (17.75)	9 (23.68)	89 (17.32)	0.440	24 (21.05)	9 (23.68)	15 (19.74)	0.807
Diabetes	50 (9.06)	5 (13.16)	45 (8.75)	0.535	15 (13.16)	5 (13.16)	10 (13.16)	1.000
Nutritional targets, mean ± SD								
Daily caloric prescription, kcal/kg/day	1.3 ± 0.2	1.3 ± 0.1	1.3 ± 0.2	0.784	1.3 ± 0.1	1.3 ± 0.1	1.3 ± 0.1	0.880
Daily protein prescription, g/kg/day	25.0 ± 0.6	25.0 ± 0.5	25.2 ± 0.6	0.686	25.1 ± 0.5	25.0 ± 0.5	25.1 ± 0.6	0.764
Enteral nutrition type, *n* (%)								
Ensure	37 (6.7)	4 (10.53)	33 (6.42)	0.522	8 (7.02)	4 (10.53)	4 (5.26)	0.517
Peptamen Junior	34 (6.16)	2 (5.26)	32 (6.23)	0.911	3 (2.63)	2 (5.26)	1 (1.32)	0.535
Fresubin Diabetes	369 (66.85)	23 (60.53)	346 (67.32)	0.497	73 (64.04)	23 (60.53)	50 (65.79)	0.730
Others	125 (22.64)	11 (28.95)	114 (22.18)	0.447	33 (28.95)	11 (28.95)	22 (28.95)	1.000

^‡^: surgical patients were defined as immediate post-surgery or major surgery within 48 h prior to ICU admission. ^∫^: patients who received these therapies within 24 h of the first dose of prokinetic drug. ^∫∫^: including sufentanil, fentanyl, or dezocine. ^†^: including intracerebral hemorrhage, subarachnoid hemorrhage, and subdural hematoma.

**Table 2:** Main results for primary and secondary outcomes.

	Unmatched Cohort				Matched Cohort			
	All (*n* = 552)	M Group (*n* = 38)	D Group (*n* = 514)	*p*	All (*n* = 114)	M-M Group (*n* = 38)	D-M Group (*n* = 76)	*p*
Primary outcomes								
Average DPP% > 80%, *n* (%)	224 (40.58)	8 (21.05)	216 (42.02)	0.018	39 (34.21)	8 (21.05)	31 (40.79)	0.034
Second outcomes								
Recurrence of EFI, *n* (%)	91 (16.49)	10 (26.32)	81 (15.76)	0.143	22 (19.3)	10 (26.32)	12 (15.79)	0.275
ICU Mortality, *n* (%)	208 (37.68)	10 (26.32)	198 (38.52)	0.185	38 (33.33)	10 (26.32)	28 (36.84)	0.361
ICU Mortality aged > 60, *n* (*n*/total)	99 (99/212)	7 (7/18)	92 (92/194)	0.488	18 (18/53)	7 (7/18)	11 (11/35)	0.587
ICU LOS, Day, mean ± SD	19.86 ±2 3.44	18.70 ± 16.43	19.95 ± 23.89	0.665	24.06 ± 38.21	18.70 ± 16.43	26.7 3± 45.21	0.292
Hospital LOS, Day, mean ± SD	30.66 ± 28.35	28.63 ± 16.43	30.81 ± 29.04	0.649	37.13 ± 42.12	28.63 ± 16.43	41.3 8± 49.84	0.128
Hospital cost, CHY (×10^5^), mean ± SD	1.91 ± 1.28	1.67 ± 1.08	1.93 ± 1.29	0.234	1.79 ± 1.35	1.67 ± 1.08	1.85 ± 1.48	0.508
Ventilation-free days ^††^, Day, mean ± SD	10.83 ± 8.68	9.16 ± 9.12	10.96 ± 8.65	0.245	11.56 ± 9.29	9.16 ± 9.12	12.76 ± 9.20	0.049
CRRT-free days ^††^, Day, mean ± SD	27.3 ± 2.95	27.5 ± 2.47	27.28 ± 2.99	0.658	27.4 ± 3.19	27.5 ± 2.47	27.36 ± 3.51	0.820
Vasopressor-free days ^††^, Day, mean ± SD	25.33 ± 4.77	25.97 ± 4.02	25.28 ± 4.82	0.386	25.69 ± 4.19	25.97 ± 4.02	25.55 ± 4.29	0.615
New onset AFib, *n* (%)	28 (5.07)	4 (10.53)	24 (4.67)	0.228	7 (6.14)	4 (10.53)	3 (3.95)	0.334
Diarrhea ^#^, *n* (%)	49 (8.88)	4 (10.53)	45 (8.75)	0.940	11 (9.65)	4 (10.53)	7 (9.21)	0.911
Constipation ^#^, *n* (%)	1 (0.18)	0 (0.00)	1 (0.19)	0.786	0 (0.00)	0 (0.00)	0 (0.00)	-
Hyperglycemia ^#^, *n* (%)	511 (92.57)	34 (89.47)	477 (92.8)	0.664	103 (90.35)	34 (89.47)	69 (90.79)	0.911
Elevated creatine kinase ^#^, *n* (%)	273 (49.46)	10 (26.32)	263 (51.17)	0.005	33 (28.95)	10 (26.32)	23 (30.26)	0.827
Elevated cardiac troponin T ^#^, *n* (%)	255 (46.2)	19 (50.0)	236 (45.91)	0.750	67 (58.77)	19 (50.0)	48 (63.16)	0.253
Hyperkalemia ^#^, *n* (%)	29 (5.25)	1 (2.63)	28 (5.45)	0.708	3 (2.63)	1 (2.63)	2 (2.63)	1.000
Hypokalemia ^#^, *n* (%)	300 (54.35)	20 (52.63)	280 (54.47)	0.959	58 (50.88)	20 (52.63)	38 (50.0)	0.947
Hypermagnesemia ^#^, *n* (%)	6 (1.08)	0 (0.00)	6 (1.17)	0.503	1 (0.87)	0 (0.00)	1 (1.32)	0.562
Hypomagnesemia ^#^, *n* (%)	167 (30.25)	7 (18.42)	160 (31.13)	0.144	15 (13.16)	7 (18.42)	8 (10.53)	0.378
Hyperphosphatemia ^#^, *n* (%)	76 (13.77)	5 (13.16)	71 (13.81)	0.896	10 (8.77)	5 (13.16)	5 (6.58)	0.413
Hypophosphatemia ^#^, *n* (%)	35 (6.34)	0 (0.00)	35 (6.81)	0.096	3 (2.63)	0 (0.00)	3 (3.95)	0.312
Delirium ^#^, *n* (%)	37 (6.70)	0 (0.00)	37 (7.19)	0.087	8 (7.01)	0 (0.00)	8 (10.53)	0.093

^††^: within 28 days of ICU stay. ^#^: within 7 days (observational period) after administration of prokinetics. Average DPP%: average percentage of daily protein prescription; EFI: enteral feeding intolerance; LOS: Length of stay; SD: standard deviation; CHY: Chinese Dollar (Yuan); AF: Atrial fibrillation.

**Table 3:** Multivariate backward logistic regression model for primary outcome in unmatched cohorts.

Variable	Unmatched Cohort			
	Feeding Success, (%)		Odds Ratio (95% CI)	*p*
	No	Yes		
EFI treatment				
Metoclopramide	30 (78.95)	8 (21.05)	1 [Reference]	0.008
Domperidone	298 (57.98)	216 (42.02)	3.001 (1.334–6.755)	
Feeding start time after admitted to ICU				
Within 7 day	294 (57.98)	213 (42.02)	1 [Reference]	0.063
≥7 day	34 (75.56)	11 (24.44)	0.505 (0.246–1.039)	
Opioid ^∫∫^				
No	99 (68.27)	46 (31.72)	1 [Reference]	0.011
Yes	229 (56.26)	178 (43.73)	1.723 (1.132–2.263)	
Placement of nasoenteric tube ^∫∫^				
No	264 (57.52)	195 (42.48)	1 [Reference]	0.016
Yes	64 (68.82)	29 (31.18)	0.545 (0.334–0.892)	
Gender				
Female	98 (53.55)	85 (46.45)	1 [Reference]	0.056
Male	230 (62.33)	139 (37.67)	0.698 (0.482–1.009)	

^∫∫^: within the observational period. EFI: enteral feeding intolerance; ICU: intensive care unit.

**Table 4:** Multivariate backward logistic regression model for primary outcome in matched cohorts.

Variable	Matched Cohort			
	Feeding Success, (%)		Odds Ratio (95% CI)	*p*
	No	Yes		
EFI treatment				
Metoclopramide	30 (78.95)	8 (21.05)	1 [Reference]	0.031
Domperidone	45 (59.21)	31 (40.89)	2.745 (1.094–6.888)	
Opioid ^∫∫^				
No	32 (76.19)	10 (23.81)	1 [Reference]	0.060
Yes	43 (58.58)	29 (38.16)	2.309 (0.966–5.522)	

^∫∫^: within the observational period. EFI: enteral feeding intolerance; BMI: Body mass index.

## Supplementary Materials

The following are available online at https://www.mdpi.com/article/10.3390/jpm11090846/s1, Table S1: Variables of patients enrolled in the study, Table S2: Biochemical test results at ICU admission, Table S3 Biochemical test results the day before prokinetic drug administration and enteral nutrition type at time of prokinetic drug administration, Table S4: Treatments during the study period, Figure S1: Percentage of patients who reached the 80% goal of protein or calories during observational period in the unmatched cohort (A,C) and in the 1:2 propensity score matched cohort (B,D) based on Model 1 as detailed in the methods, Figure S2: The amount of enteral nutrition volume, calories, and protein delivered during observational period in the unmatched cohort (A,C,E) and the 1:2 propensity score matched cohort (B,D,F) based on Model 1 as detailed in the methods, Table S5: Univariate regression model for primary outcome in unmatched and matched cohorts, Table S6 Primary outcomes by propensity score matching using different ratios based on Model 1, Figure S3: Percentage of daily protein prescription in protein goal (DPP%) through the observational period in (A) 1:1, (B) 1:3, (C) 1:4, and (D) 1:5 propensity score matched cohorts based on Model 1 as detailed in the methods, Table S7: Primary outcomes by propensity score matching using different ratios based on Model 2, Figure S4: Percentage of daily protein prescription in protein goal (DPP%) through the observational period in (A) 1:1, (B) 1:2, (C) 1:3, and (D) 1:4 propensity score matched cohorts based on Model 2 as detailed in the methods.

## Abbreviations

EFI	enteral feeding intolerance
average DPP%	average percentage of daily protein prescription
ICU	intensive care unit
IV	intravenous
STROBE	Strengthening the Reporting of Observational studies in Epidemiology
average DPP%	average percentage of daily protein prescription
M group	metoclopramide group
D group	domperidone group
M-M group	metoclopramide group after propensity score matching
D-M group	domperidone group after propensity score matching
DPP%	daily protein prescription in protein goal
