# Supplementary material for: Association between Domperidone Administered via Feeding Tube and Feeding Success in Critically Ill Patients with Enteral Feeding Intolerance
Source: J Pers Med. 2021 Aug 27;11(9):846. doi: 10.3390/jpm11090846 (PMC8466316; doi:10.3390/jpm11090846)

Supplementary Methods

Definition of primary outcome and secondary outcomes

In our department, a volume-based feeding protocol was employed. The target of protein for each patient was 1.3 g/kg estimated dry body weight per day, and the target of calorie was 25 kcal/kg estimated dry body weight per day. For patients with BMI ≥ 30, ideal body weight was used to determine the target for protein and calories. Daily protein prescription (DPP) was defined as protein delivered by enteral nutrition. DPP % was defined as the ratio between DPP and the target of protein. The average DPP % was calculated using the following formula: average DPP % = $(\frac{\sum_{day1}^{day7}(\mathrm{DPP})}{7})/(1.3 * \text{body weight})$. The daily calories were delivered by enteral nutrition (the calories of propofol were not included).

Recurrence of EFI was defined as any episode of EFI after prokinetic agent administration. New onset atrial fibrillation was scanned according to diagnosis when discharged from the ICU. Diarrhea was defined as more than 3 bowel movements per day and stool weight greater than 200 grams per day or volume greater than 250 mL per day which needed to be interrupted. Constipation was defined as failure of the bowel to open for three consecutive days. Hyperglycemia was defined as any episode of blood glucose > 7.2 mmol/L. Elevated creatine kinase was defined as any episode of creatine kinase level > 198 IU/L, while the elevated cardiac troponin T was defined as any episode of cardiac troponin T level > 0.5 μg/L. Hyperkalemia and hypokalemia were defined as blood potassium level > 5.5 mmol/L or < 3.5 mmol/L, respectively. Hypermagnesemia and hypomagnesemia were defined as blood magnesia level > 1.5 mmol/L or < 0.75 mmol/L, respectively. Hyperphosphatemia and hypophosphatemia were defined as blood phosphoremia level > 1.46 mmol/L or < 0.32 mmol/L, respectively. Delirium was defined according to conventional widely accepted standards based on Confusion Assessment Method for the ICU (CAM-ICU) method. All the events were determined through patients' personal record review by two independent intensivists.

Covariates, missing value imputation, and statistical methods

All covariates included in our study are listed below, with the percentage of missing values. For any missing lab values, we used the mean value of the entire cohort at that same time point as the imputed value. The results of lab test at admission were defined as the worst value obtained within 24 hours after admission. The results of lab test before starting prokinetic agents were defined as the worst value obtained within 24 hours before prokinetic agent starting.

Descriptive statistics were used to characterize the study population. Continuous variables were reported as medians with interquartile ranges or means with standard deviation, and categorical variables were reported as counts with relative frequencies.

Categorical data were compared with χ^2 test and continuous data with the Wilcoxon rank sum test.

Univariate logistic regression analysis was performed to assess the independent association of clinical factors, including prokinetic agents, feeding start time, supplementary parenteral nutrition, prokinetic agent, administration of other agents (insulin, probiotic, opioid, propofol, muscle relaxant, and vasopressor), CRRT, placement of nasoenteric tube, disease severity, age, gender, admission type, BMI, and hospitalization time before ICU admission, and feeding success. Multivariate logistic regression analyses were fitted to examine association for prokinetic agents and feeding success. All potential confounders using in univariate logistic regression analysis were introduced as candidate covariates using backward elimination logistic regression method. We report the results from the regression model as odds ratios with 95% confidence intervals.

The propensity score was calculated with 1:1 matching to 1:5 matching because of limited patient numbers in the metoclopramide group using nearest neighbor matching. Potential confounders in the matching model were selected based on logistic regression and clinical interests. In Model 1, we matched patients based on baseline factors of clinical interest and lab results before prokinetic treatment with $P < 0.10$: age (as a continues variable), gender (as a categorical variable), admission type (as a categorical variable), APACHE score (as a continuous variable), comorbidity (as a categorical variable), ICU admission diagnosis (as a categorical variable), BMI (as a continuous variable), CRP (as a continuous variable), TT (as a continuous variable), ATIII (as a continuous variable), ALT (as a continuous variable), AST (as a continuous variable), TB (as a continuous variable), HBDB (as a continuous variable), GLU (as a continuous variable), LDH (as a continuous variable), ALP (as a continuous variable), CHOL (as a continuous variable), TP (as a continuous variable), GLB (as a continuous variable), DBIL (as a continuous variable), and PLT (as a continuous variable).
In Model 2, we matched patients based on baseline factors of clinical interest and lab results at admission with $P < 0.10$: age (as a continuous variable), gender (as a categorical variable), admission type (as a categorical variable), APACHE score (as a continuous variable), comorbidity (as a categorical variable), ICU admission diagnosis (as a categorical variable), BMI (as a continuous variable), APTT (as a continuous variable), TB (as a continuous variable), IBIL (as a continuous variable), HBDH (as a continuous variable), TP (as a continuous variable), GLB (as a continuous variable), GGT (as a continuous variable), PH (as a continuous variable), HCO_3 (as a continuous variable), DBIL (as a continuous variable), and PLT (as a continuous variable).
One-to-one nearest neighbor matching was performed between groups without replacement using a caliper width of 0.20 times of the standard deviation of the logit of the propensity score. To assess the performance of the matching, we compared baseline variables between the matched groups using method mentioned above. In the matched cohort, we performed logistic regression, chi-square test, Fisher's exact test and McNemar's test to assess the association between prokinetic agents and feeding success

when it is appropriate.

Our statistical analysis tools included: pandas (0.23.4), numpy (1.15.1), matplotlib (2.2.3), xlrd (1.1.0), ctmatching (0.0.6), scipy (1.1.10), itertools (4.3.0), scikit-learn (0.19.2), and seaborn (0.9.0).

Table S1: Variables of patients enrolled in the study

Variables	Missing percentage (%)
Age	0.00
Gender	0.00
BMI	0.00
APACHE II	0.00
SOFA	0.00
NUTRIC score	0.00
Admission type	0.00
Admission reason	0.00
Time before ICU admission	0.00
Feeding start after admission	0.00
Prokinetics start time	0.00
Clinical situation when prokinetics started	0.00
ICU admission diagnosis	0.00
Comorbidity	0.00
CRP at admission	5.72
PCT at admission	7.25
IL-6 at admission	5.07
INR at admission	0.36
PT at admission	0.36
TT at admission	0.36
ATIII at admission	0.91
APTT at admission	0.36
Fib, at admission	0.36
D-dimer at admission	0.91
ALT at admission	0.18
AST at admission	0.18
TB at admission	0.18
IB at admission	0.18
TBA at admission	7.97
LDH at admission	0.18
Urea at admission	0.18
Uric acid at admission	0.18
Pro-BNP at admission	6.74
HBDH at admission	0.18
CK at admission	0.18
TPN-T at admission	7.28
Glu at admission	0.00

ALP at admission	0.18
TP at admission	0.18
GLB at admission	0.18
ALB at admission	0.18
DBIL at admission	0.18
Cys-c at admission	0.18
GGT at admission	0.18
CHOL at admission	0.18
TG at admission	0.18
LDL-C at admission	0.18
HDL-C at admission	0.18
Lac at admission	1.45
pH at admission	0.36
PaO_2 at admission	0.91
$PaCO_2$ at admission	0.91
HCO_3 at admission	4.17
AG at admission	0.00
RBC at admission	0.00
HCT at admission	0.00
WBC at admission	0.00
MCH at admission	0.00
MCHC at admission	0.00
PLT at admission	0.00
HGB at admission	0.00
Na at admission	0.00
K at admission	0.00
Ca at admission	0.00
Mg at admission	0.00
P at admission	0.00
Cl at admission	0.00
CRP the day before prokinetic drug administration	5.65
PCT the day before prokinetic drug administration	5.65
IL-6 the day before prokinetic drug administration	6.74
INR the day before prokinetic drug administration	8.99
PT the day before prokinetic drug administration	8.99
TT the day before prokinetic drug administration	9.71
ATIII the day before prokinetic drug administration	9.42
APTT the day before prokinetic drug administration	9.53
Fib, the day before prokinetic drug administration	9.71
D-Dimer the day before prokinetic drug administration	9.6
ALT the day before prokinetic drug administration	9.31
AST the day before prokinetic drug administration	9.31
TB the day before prokinetic drug administration	9.31
IB the day before prokinetic drug administration	9.31

TBA the day before prokinetic drug administration	5.36
LDH the day before prokinetic drug administration	8.37
UREA the day before prokinetic drug administration	9.31
URIC the day before prokinetic drug administration	9.31
Pro-BNP the day before prokinetic drug administration	5.4
HBDH the day before prokinetic drug administration	8.37
CK the day before prokinetic drug administration	8.37
TPN-T the day before prokinetic drug administration	8.12
Glu the day before prokinetic drug administration	2.17
ALP the day before prokinetic drug administration	9.31
TP the day before prokinetic drug administration	9.31
GLB the day before prokinetic drug administration	9.31
ALB the day before prokinetic drug administration	9.31
DBIL the day before prokinetic drug administration	9.31
Cys-c the day before prokinetic drug administration	9.31
GGT the day before prokinetic drug administration	9.31
CHOL the day before prokinetic drug administration	7.46
TG the day before prokinetic drug administration	7.46
LDL-C the day before prokinetic drug administration	7.46
HDL-C the day before prokinetic drug administration	7.46
Lac the day before prokinetic drug administration	9.42
pH the day before prokinetic drug administration	6.16
PaO_2 the day before prokinetic drug administration	8.88
$PaCO_2$ the day before prokinetic drug administration	9.06
HCO_3 the day before prokinetic drug administration	8.73
AG the day before prokinetic drug administration	6.88
RBC the day before prokinetic drug administration	8.04
HCT the day before prokinetic drug administration	8.04
WBC the day before prokinetic drug administration	8.04
MCH the day before prokinetic drug administration	8.04
MCHC the day before prokinetic drug administration	8.04
PLT the day before prokinetic drug administration	8.04
HGB the day before prokinetic drug administration	8.04
Na the day before prokinetic drug administration	1.27
K the day before prokinetic drug administration	1.27
Mg the day before prokinetic drug administration	6.45
P the day before prokinetic drug administration	6.45
Cl the day before prokinetic drug administration	5.8
Enteral nutrition type	0.00
Sufentanil	0.00
Fentanyl	0.00
Dezocine	0.00
Propofol	0.00
Probiotic	0.00

Muscle relaxants	0.00
Mosapride	0.00
Fat Emulsion Injection	0.00
Insulin	0.00
Parenteral nutrition (Kabiven)	0.00
ICU LOS	0.00
Hospital LOS	0.00
Hospital cost	0.00
ICU Mortality	0.00
Recurrence of EFI	0.00
New onset AFib	0.00
Ventilation-free days	0.00
CRRT-free days	0.00
Vasopressor-free days	0.00
Diarrhea	0.00
Constipation	0.00
Hyperglycemia	0.00
Delirium	0.00

SD: standard deviation; CRP: C-reactive protein; PCT: Procalcitonin; PT: prothrombin time; TT: Thrombin time; AT III: Antithrombin III; APTT: Activated partial thromboplastin time; Fib: fibrinogen; ALT: Alanine Aminotransferase; AST: Aspartate Aminotransferase; TB: Total bilirubin; IB: Indirect bilirubin; TBA: Total bile acids; LDH: Lactate dehydrogenase; BNP: type B natriuretic peptide; HBDH: hydroxybutyrate dehydrogenase; CK: Creatine kinase; cTNT: cardiac troponin T; Glu: Glucose; ALP: Alkaline Phosphatase; Cys-c: Cystatin C; GGT: Gamma-Glutamyl Transferase; CHOL: cholesterol; TG: Triglycerides; LDL-C: Low-density lipoprotein cholesterol; HDL-C: High-density lipoprotein cholesterol; LAC: lactate; AG: anion gap; RBC: Red blood cell; HCT: Hematocrit; WBC: white blood cell; MCH: Mean corpuscular hemoglobin; MCHC: Mean corpuscular hemoglobin concentration; PLT: platelet; HGB: Hemoglobin;

Table S2 Biochemical test results at ICU admission

	Unmatched cohort				Matched cohort†			
	All (n=552)	M group (n=38)	D group (n=514)	*P*	All (n=114)	M-M group (n=38)	D-M group (n=76)	*P*
Inflammatory marker, mean ± SD								
CRP, mg/L	83.20 ± 85.22	75.31 ± 63.62	83.80 ± 86.67	0.606	80.88 ± 84.61	75.31 ± 63.62	83.40 ± 92.93	0.672
PCT, ng/mL	5.26 ± 14.32	4.44 ± 11.53	5.32 ± 14.51	0.731	2.69 ± 8.20	4.44 ± 11.53	1.89 ± 6.02	0.133
IL-6, pg/mL	310.21 ± 791.48	433.43 ± 1020.05	300.95 ± 772.59	0.402	277.31 ± 677.62	433.43 ± 1020.05	208.22 ± 446.19	0.152
Coagulation test, mean ± SD								
INR	1.34 ± 0.56	1.21 ± 0.25	1.35 ± 0.57	0.135	1.21 ± 0.43	1.21 ± 0.25	1.22 ± 0.50	0.977
PT, s	15.72 ± 6.31	14.19 ± 2.93	15.83 ± 6.48	0.121	14.24 ± 4.95	14.19 ± 2.93	14.26 ± 5.71	0.941
TT, s	19.72 ± 8.36	19.75 ± 9.33	19.71 ± 8.30	0.980	19.76 ± 11.09	19.75 ± 9.33	19.76 ± 11.93	0.997
ATIII, %	62.11 ± 25.11	66.68 ± 17.68	61.78 ± 25.55	0.252	70.17 ± 18.44	66.68 ± 17.68	71.89 ± 18.67	0.154
APTT, s	44.16 ± 23.37	37.00 ± 10.40	44.69 ± 23.97	0.050	36.18 ± 9.23	37.0 ± 10.40	35.77 ± 8.63	0.505
Fib, g/L	3.14 ± 1.71	3.07 ± 1.70	3.14 ± 1.71	0.793	3.31 ± 1.60	3.07 ± 1.70	3.44 ± 1.54	0.264
D-dimer, mg/L FEU	7.58 ± 7.57	7.23 ± 6.31	7.61 ± 7.66	0.732	6.46 ± 6.20	7.23 ± 6.31	6.08 ± 6.16	0.359
Biochemical analysis, mean ± SD								
ALT, IU/L	142.99 ± 410.68	43.76 ± 87.34	150.34 ± 424.07	0.123	39.68 ± 62.57	43.76 ± 87.34	37.64 ± 46.07	0.625
AST, IU/L	292.07 ± 972.55	44.55 ± 55.17	310.41 ± 1005.46	0.104	46.16 ± 67.81	44.55 ± 55.17	46.96 ± 73.64	0.845
TB, µmol/L	31.95 ± 49.02	16.94 ± 13.15	33.06 ± 50.51	0.050	14.48 ± 10.15	16.94 ± 13.15	13.25 ± 8.09	0.067
IB, µmol/L	9.98 ± 15.06	6.44 ± 3.11	10.24 ± 15.56	0.134	5.99 ± 3.21	6.44 ± 3.11	5.76 ± 3.25	0.282
TBA, µmol/L	20.68 ± 50.39	7.10 ± 13.97	22.25 ± 52.80	0.163	5.12 ± 10.16	7.10 ± 13.97	3.22 ± 3.43	0.185
LDH, mmol/L	546.92 ± 929.12	274.47 ± 187.81	567.10 ± 958.58	0.061	292.20 ± 272.45	274.47 ± 187.81	301.07 ± 306.91	0.625
Urea, mmol/L	9.05 ± 6.73	9.44 ± 7.61	9.02 ± 6.67	0.711	8.19 ± 6.90	9.44 ± 7.61	7.56 ± 6.49	0.172
Uric acid, µmol/L	240.22 ± 137.26	240.66 ± 113.83	240.19 ± 138.94	0.984	232.09 ± 120.03	240.66 ± 113.83	227.81 ± 123.53	0.592

Pro-BNP, pg/ml	3194.12 ± 7021.3	2760.39 ± 6513.39	3233.56 ± 7072.05	0.699	2666.63 ± 6960.42	2760.39 ± 6513.39	2617.71 ± 7228.63	0.919
HBDH, IU/L	350.66 ± 414.35	217.92 ± 143.08	360.49 ± 426.08	0.041	227.26 ± 161.13	217.92 ± 143.08	231.93 ± 170.15	0.645
CK, IU/L	754.84 ± 2538.44	397.16 ± 583.32	781.33 ± 2624.33	0.368	458.26 ± 1172.21	397.16 ± 583.32	488.82 ± 1378.25	0.696
cTNT, ng/L	98.18 ± 353.78	62.68 ± 97.09	101.34 ± 367.93	0.536	60.43 ± 101.67	62.68 ± 97.09	59.25 ± 104.68	0.872
Glu, mmol/L	9.14 ± 3.42	8.90 ± 2.76	9.16 ± 3.46	0.642	8.59 ± 2.77	8.90 ± 2.76	8.44 ± 2.78	0.414
ALP, IU/L	84.69 ± 67.53	69.50 ± 40.42	85.81 ± 69.01	0.151	69.18 ± 38.85	69.50 ± 40.42	69.03 ± 38.32	0.951
TP, g/L	53.05 ± 9.15	55.97 ± 8.37	52.83 ± 9.17	0.041	54.57 ± 9.25	55.97 ± 8.37	53.87 ± 9.63	0.255
GLB, g/L	21.66 ± 6.72	23.41 ± 5.88	21.53 ± 6.76	0.096	23.24 ± 6.19	23.41 ± 5.88	23.15 ± 6.38	0.834
ALB, g/L	31.46 ± 6.10	32.56 ± 5.44	31.38 ± 6.14	0.249	31.33 ± 5.68	32.56 ± 5.44	30.71 ± 5.72	0.102
Cys-c, mg/L	1.27±0.97	1.30 ± 1.19	1.27 ± 0.95	0.844	1.24 ± 1.19	1.30 ± 1.19	1.21 ± 1.20	0.716
GGT, IU/L	71.15 ± 125.75	37.24 ± 33.69	73.66 ± 129.66	0.085	47.40 ± 61.17	37.24 ± 33.69	52.49 ± 70.7	0.211
Chol, mmol/L	2.71 ± 1.15	2.76 ± 0.95	2.70 ± 1.17	0.775	3.00 ± 1.06	2.76 ± 0.95	3.12 ± 1.09	0.081
TG, mmol/L	1.40 ± 1.10	1.45 ± 1.13	1.40 ± 1.10	0.792	1.37 ± 0.93	1.45 ± 1.13	1.33 ± 0.83	0.529
LDL-C, mmol/L	1.34 ± 0.81	1.31 ± 0.71	1.35 ± 0.82	0.805	1.52 ± 0.82	1.31 ± 0.71	1.62 ± 0.86	0.057
HDL-C, mmol/L	0.69 ± 0.44	0.73 ± 0.47	0.69 ± 0.44	0.545	0.83 ± 0.51	0.73 ± 0.47	0.88 ± 0.52	0.154
Lac, mmol/L	2.07 ± 1.60	1.89 ± 1.04	2.08 ± 1.64	0.493	1.98 ± 1.40	1.89 ± 1.04	2.03 ± 1.55	0.626
Arterial Blood Gas Test, mean ± SD								
pH	7.27 ± 0.49	7.14 ± 0.59	7.28 ± 0.48	0.086	7.18 ± 0.63	7.14 ± 0.59	7.21 ± 0.65	0.569
PaO_2, mmHg	103.18 ± 44.88	110.01 ± 41.83	102.7 ± 45.08	0.352	106.32 ± 53.01	110.01 ± 41.83	104.57 ± 57.73	0.619
$PaCO_2$, mmHg	39.33 ± 9.26	36.89 ± 5.20	39.51 ± 9.46	0.102	38.32 ± 8.25	36.89 ± 5.20	39.01 ± 9.32	0.207
HCO_3, mmol/L	24.79 ± 5.14	23.38 ± 3.52	24.89 ± 5.22	0.093	24.72 ± 5.15	23.38 ± 3.52	25.34 ± 5.67	0.063
AG, mmol/L	11.64 ± 6.99	10.49 ± 4.79	11.72 ± 7.12	0.306	12.21 ± 6.02	10.49 ± 4.79	13.07 ± 6.41	0.055
Complete blood count, mean ± SD								
RBC, $\times 10^{12}$/L	3.39 ± 0.79	3.35 ± 0.62	3.39 ± 0.8	0.762	3.50 ± 0.79	3.35 ± 0.62	3.58 ± 0.86	0.145
HCT, L/L	0.31 ± 0.07	0.30 ± 0.06	0.31 ± 0.07	0.604	0.32 ± 0.07	0.30 ± 0.06	0.33 ± 0.07	0.074
WBC, $\times 10^9$/L	11.35 ± 5.78	11.42 ± 7.92	11.34 ± 5.60	0.934	11.48 ± 6.04	11.42 ± 7.92	11.50 ± 4.91	0.948

MCH, pg	30.03 ± 2.38	29.68 ± 2.45	30.06 ± 2.37	0.358	30.04 ± 2.30	29.68 ± 2.45	30.22 ± 2.22	0.251
MCHC, g/L	326.20 ± 15.22	324.87 ± 17.32	326.30 ± 15.06	0.576	325.78 ± 15.55	324.87 ± 17.32	326.24 ± 14.69	0.660
PLT, $\times 10^9$/L	133.21 ± 92.72	144.18 ± 67.31	132.39 ± 94.33	0.450	157.76 ± 84.43	144.18 ± 67.31	164.55 ± 91.46	0.226
HGB, g/L	101.16 ± 22.93	99.05 ± 19.35	101.31 ± 23.18	0.558	104.68 ± 23.12	99.05 ± 19.35	107.49 ± 24.43	0.066
Na, mmol/L	139.77 ± 6.91	139.84 ± 6.7	139.77 ± 6.93	0.953	140.14 ± 7.67	139.84 ± 6.7	140.29 ± 8.15	0.766
K, mmol/L	3.87 ± 0.53	3.90 ± 0.58	3.87 ± 0.52	0.778	3.92 ± 0.52	3.90 ± 0.58	3.92 ± 0.50	0.799
Ca, mmol/L	2.03 ± 0.22	2.11 ± 0.22	2.02 ± 0.22	0.027	2.06 ± 0.21	2.11 ± 0.22	2.04 ± 0.21	0.134
Mg, mmol/L	0.85 ± 0.23	0.88 ± 0.16	0.85 ± 0.24	0.432	0.86 ± 0.17	0.88 ± 0.16	0.85 ± 0.17	0.445
P, mmol/L	0.95 ± 0.42	0.95 ± 0.38	0.95 ±0 .42	0.908	0.98 ± 0.39	0.95 ± 0.38	0.99 ± 0.39	0.627
Cl, mmol/L	108.55 ± 7.57	110.29 ± 7.94	108.42 ± 7.53	0.152	108.74 ± 8.74	110.29 ± 7.94	108.0 ± 9.06	0.197

†: 1 to 2 propensity score matching based on model 1 as described in method

SD: standard deviation; CRP: C-reactive protein; PCT: Procalcitonin; PT: prothrombin time; TT: Thrombin time; AT III: Antithrombin III; APTT: Activated partial thromboplastin time; Fib: fibrinogen; ALT: Alanine Aminotransferase; AST: Aspartate Aminotransferase; TB: Total bilirubin; IB: Indirect bilirubin; TBA: Total bile acids; LDH: Lactate dehydrogenase; BNP: type B natriuretic peptide; HBDH: hydroxybutyrate dehydrogenase; CK: Creatine kinase; cTNT: cardiac troponin T; Glu: Glucose; ALP: Alkaline Phosphatase; Cys-c: Cystatin C; GGT: Gamma-Glutamyl Transferase; CHOL: cholesterol; TG: Triglycerides; LDL-C: Low-density lipoprotein cholesterol; HDL-C: High-density lipoprotein cholesterol; LAC: lactate; AG: anion gap; RBC: Red blood cell; HCT: Hematocrit; WBC: white blood cell; MCH: Mean corpuscular hemoglobin; MCHC: Mean corpuscular hemoglobin concentration; PLT: platelet; HGB: Hemoglobin;

Table S3 Biochemical test results the day before prokinetic drug administration and enteral nutrition type at time of prokinetic drug administration

	Unmatched cohort				Matched cohort†			
	All (n = 552)	M group (n = 38)	D group (n = 514)	*P*	All (n = 114)	M-M group (n = 38)	D-M group (n = 76)	*P*
Enteral nutrition type, n (%)								
Ensure	37 (6.7)	4 (10.53)	33 (6.42)	0.522	8 (7.02)	4 (10.53)	4 (5.26)	0.517
Peptamen Junior	34 (6.16)	2 (5.26)	32 (6.23)	0.911	3 (2.63)	2 (5.26)	1 (1.32)	0.535
Fresubin Diabetes	369 (66.85)	23 (60.53)	346 (67.32)	0.497	73 (64.04)	23 (60.53)	50 (65.79)	0.730
Others	125 (22.64)	11 (28.95)	114 (22.18)	0.447	33 (28.95)	11 (28.95)	22 (28.95)	1.000
Inflammatory marker, mean ± SD								
CRP, mg/L	102.89 ± 89.53	41.81 ± 48.34	107.77 ± 90.36	0.014	53.02 ± 47.38	41.81 ± 48.34	59.42 ± 46.78	0.312
PCT, ng/mL	5.23 ± 13.66	1.33 ± 1.92	5.50 ± 14.07	0.199	0.92 ± 1.95	1.33 ± 1.92	0.74 ± 1.96	0.269
Il-6, pg/mL	312.49 ± 831.72	176.39 ± 338.54	323.83 ± 859.83	0.557	120.68 ± 232.23	176.39 ± 338.54	85.49 ± 128.33	0.296
Coagulation test, mean ± SD								
INR	1.38 ± 0.58	1.22 ± 0.28	1.39 ± 0.60	0.168	1.23 ± 0.53	1.22 ± 0.28	1.24 ± 0.63	0.875
PT, s	16.09 ± 6.62	14.19 ± 3.28	16.22 ± 6.77	0.146	14.40 ± 6.02	14.19 ± 3.28	14.52 ± 7.17	0.829
TT, s	19.76 ± 9.26	23.08 ± 21.29	19.55 ± 7.93	0.076	21.28 ± 17.84	23.08 ± 21.29	20.30 ± 15.84	0.553
ATIII, %	62.28 ± 26.63	73.82 ± 21.07	61.64 ± 26.78	0.052	74.59 ± 18.43	73.82 ± 21.07	74.97 ± 17.25	0.826
APTT, s	45.11 ± 24.39	40.90 ± 31.28	45.38 ± 23.92	0.393	37.59 ± 20.56	40.90 ± 31.28	35.78 ± 11.21	0.341
Fib, g/L	3.29 ± 1.89	3.18 ± 1.65	3.30 ± 1.90	0.766	3.73 ± 1.69	3.18 ± 1.65	4.04 ± 1.65	0.048
D-dimer, mg/L FEU	7.67 ± 7.60	7.31 ± 6.36	7.69 ± 7.67	0.804	7.05 ± 6.47	7.31 ± 6.36	6.92 ± 6.60	0.829
Biochemical analysis, mean ± SD								
ALT, IU/L	141.11 ± 324.01	34.16 ± 33.94	148.61 ± 333.8	0.057	35.27 ± 31.17	34.16 ± 33.94	35.84 ± 29.95	0.809
AST, IU/L	263.76 ± 792.44	36.68 ± 34.61	279.68 ± 817.4	0.099	40.91 ± 36.11	36.68 ± 34.61	43.07 ± 36.94	0.426
TB, μmol/L	34.66 ± 51.69	15.27 ± 11.59	36.02 ± 53.13	0.031	13.48 ± 9.82	15.27 ± 11.59	12.57 ± 8.75	0.215

IB, μmol/L	9.10 ± 13.08	5.66 ± 3.10	9.34 ± 13.48	0.131	5.74 ± 3.01	5.66 ± 3.10	5.78 ± 2.99	0.868
TBA, μmol/L	22.73 ± 49.38	6.09 ± 8.19	24.19 ± 51.21	0.245	4.86 ± 6.29	6.09 ± 8.19	3.89 ± 4.36	0.397
LDH, mmol/L	550.19 ± 869.47	264.83 ± 129.14	567.35 ± 891.85	0.098	278.69 ± 120.14	264.83 ± 129.14	285.62 ± 116.17	0.493
UREA, mmol/L	10.58 ± 9.04	9.03 ± 7.75	10.68 ± 9.12	0.326	7.99 ± 5.86	9.03 ± 7.75	7.46 ± 4.6	0.225
URIC, μmol/L	210.38 ± 130.85	200.83 ± 132.36	211.05 ± 130.87	0.680	176.48 ± 111.78	200.83 ± 132.36	164.11 ± 98.63	0.137
Pro-BNP, pg/ml	4241.13 ± 7758.26	2057.07 ± 2145.67	4413.88 ± 8014.3	0.275	2815.41 ± 7141.34	2057.07 ± 2145.67	3147.19 ± 8469.15	0.639
HBDH, IU/L	364.33 ± 429.96	219.17 ± 116.66	373.06 ± 440.31	0.089	227.65 ± 106.73	219.17 ± 116.66	231.9 ± 102.44	0.637
CK, IU/L	606.18 ± 1942.88	292.54 ± 555.94	625.04 ± 1994.55	0.416	235.12 ± 379.56	292.54 ± 555.94	206.42 ± 252.7	0.368
cTNT, ng/L	130.89 ± 405.42	60.98 ± 103.42	137.88 ± 423.5	0.471	48.54 ± 84.65	60.98 ± 103.42	41.43 ± 72.97	0.468
Glu, mmol/L	9.29 ± 3.35	8.33 ± 2.53	9.36 ± 3.40	0.069	8.63 ± 2.92	8.33 ± 2.53	8.79 ± 3.10	0.440
ALP, IU/L	113.84 ± 102.03	84.42 ± 42.63	115.9 ± 104.66	0.097	83.10 ± 39.82	84.42 ± 42.63	82.43 ± 38.65	0.822
TP, g/L	56.87 ± 9.08	61.66 ± 7.22	56.53 ± 9.11	0.002	60.02 ± 6.88	61.66 ± 7.22	59.19 ± 6.60	0.116
GLB, g/L	23.06 ± 7.23	26.94 ± 7.59	22.78 ± 7.14	0.002	25.54 ± 6.25	26.94 ± 7.59	24.83 ± 5.38	0.127
ALB, g/L	33.89 ± 5.85	34.72 ± 4.01	33.84 ± 5.96	0.416	34.48 ± 3.87	34.72 ± 4.01	34.36 ± 3.83	0.678
Cys-c, mg/L	1.41 ± 1.07	1.38 ± 1.25	1.41 ± 1.05	0.873	1.22 ± 0.96	1.38 ± 1.25	1.14 ± 0.78	0.276
GGT, IU/L	96.02 ± 158.56	55.55 ± 42.92	98.86 ± 163.28	0.142	65.39 ± 71.63	55.55 ± 42.92	70.39 ± 82.37	0.350
CHOL, mmol/L	2.69 ± 1.16	3.11 ± 1.04	2.67 ± 1.17	0.062	3.18 ± 0.9	3.11 ± 1.04	3.22 ± 0.82	0.648
TG, mmol/L	1.52 ± 1.25	1.89 ± 1.33	1.49 ± 1.24	0.128	1.60 ± 1.04	1.89 ± 1.33	1.45 ± 0.84	0.089
LDL-C, mmol/L	1.29 ± 0.81	1.55 ± 0.72	1.27 ± 0.82	0.100	1.64 ± 0.67	1.55 ± 0.72	1.69 ± 0.65	0.403
HDL-C, mmol/L	0.58 ± 0.38	0.68 ± 0.35	0.57 ± 0.39	0.150	0.75 ± 0.40	0.68 ± 0.35	0.78 ± 0.42	0.314
Lac, mmol/L	1.80 ± 1.10	1.70 ± 0.95	1.80 ± 1.11	0.615	1.68 ± 1.13	1.70 ± 0.95	1.67 ± 1.21	0.887
Arterial Blood Gas Test, mean ± SD								
pH	7.34 ± 0.37	7.35 ± 0.29	7.34 ± 0.37	0.952	7.33 ± 0.41	7.35 ± 0.29	7.32 ± 0.46	0.751
PaO_2, mmHg	99.28 ± 43.57	100.23 ± 41.14	99.21 ± 43.77	0.897	103.82 ± 56.73	100.23 ± 41.14	105.50 ± 62.88	0.612
$PaCO_2$, mmHg	39.63 ± 8.72	38.07 ± 4.99	39.74 ± 8.92	0.287	39.72 ± 8.74	38.07 ± 4.99	40.49 ± 9.96	0.189
HCO_3, mmol/L	25.49 ± 5.04	24.81 ± 3.97	25.55 ± 5.11	0.433	25.96 ± 5.10	24.81 ± 3.97	26.51 ± 5.50	0.128

AG, mmol/L	10.9 ± 6.70	10.89 ± 6.24	10.90 ± 6.73	0.991	10.88 ± 6.39	10.89 ± 6.24	10.88 ± 6.51	0.998
Complete blood count, mean±SD								
RBC, $\times 10^{12}$/L	3.26 ± 0.74	3.27 ± 0.53	3.26 ± 0.75	0.925	3.34 ± 0.72	3.27 ± 0.53	3.38 ± 0.81	0.474
HCT, L/L	0.30 ± 0.06	0.30 ± 0.05	0.30 ± 0.06	0.937	0.31 ± 0.06	0.30 ± 0.05	0.32 ± 0.07	0.277
WBC, $\times 10^9$/L	11.18 ± 5.61	10.10 ± 4.18	11.26 ± 5.69	0.261	9.95 ± 3.82	10.10 ± 4.18	9.87 ± 3.65	0.783
MCH, pg	30.07 ± 2.27	29.63 ± 2.37	30.11 ± 2.26	0.275	30.05 ± 2.15	29.63 ± 2.37	30.28 ± 2.01	0.168
MCHC, g/L	322.91 ± 15.08	321.75 ± 13.81	322.99 ± 15.18	0.654	321.84 ± 12.88	321.75 ± 13.81	321.88 ± 12.47	0.963
PLT, $\times 10^9$/L	153.74 ± 116.77	188.31 ± 102.31	151.27 ± 117.45	0.083	187.43 ± 98.59	188.31 ± 102.31	186.95 ± 97.41	0.950
HGB, g/L	97.46 ± 20.89	96.53 ± 15.29	97.52 ± 21.24	0.795	99.93 ± 19.96	96.53 ± 15.29	101.78 ± 21.99	0.233
Na, mmol/L	139.77 ± 8.13	138.53 ± 7.70	139.86 ± 8.16	0.310	140.65 ± 9.31	138.53 ± 7.7	141.72 ± 9.9	0.085
K, mmol/L	3.91 ± 0.52	3.96 ±0 .57	3.90 ± 0.52	0.496	3.91 ± 0.51	3.96 ± 0.57	3.88 ± 0.49	0.453
Mg, mmol/L	0.85 ± 0.17	0.90 ± 0.18	0.85 ± 0.17	0.132	0.88 ± 0.15	0.90 ± 0.18	0.87 ± 0.13	0.361
P, mmol/L	0.93 ± 0.41	0.94 ± 0.37	0.93 ± 0.42	0.840	0.88 ± 0.32	0.94 ± 0.37	0.84 ±0.30	0.212
Cl, mmol/L	108.0 ± 8.09	106.55 ± 9.67	108.1 ± 7.97	0.275	107.59 ± 8.68	106.55 ± 9.67	108.12 ± 8.15	0.387

†: 1 to 2 propensity score matching based on model 1 as described in methods section

SD: standard deviation; CRP: C-reactive protein; PCT: Procalcitonin; PT: prothrombin time; TT: Thrombin time; AT III: Antithrombin III; APTT: Activated partial thromboplastin time; Fib: fibrinogen; ALT: Alanine Aminotransferase; AST: Aspartate Aminotransferase; TB: Total bilirubin; IB: Indirect bilirubin; TBA: Total bile acids; LDH: Lactate dehydrogenase; BNP: type B natriuretic peptide; HBDH: hydroxybutyrate dehydrogenase; CK: Creatine kinase; cTNT: cardiac troponin T; Glu: Glucose; ALP: Alkaline Phosphatase; Cys-c: Cystatin C; GGT: Gamma-Glutamyl Transferase; CHOL: cholesterol; TG: Triglycerides; LDL-C: Low-density lipoprotein cholesterol; HDL-C: High-density lipoprotein cholesterol; LAC: lactate; AG: anion gap; RBC: Red blood cell; HCT: Hematocrit; WBC: white blood cell; MCH: Mean corpuscular hemoglobin; MCHC: Mean corpuscular hemoglobin concentration; PLT: platelet; HGB: Hemoglobin;

Table S4 Treatments during the study period

	Unmatched cohort				Matched cohort‡			
	All (n = 552)	M group (n = 38)	D group (n = 514)	*P*	All (n = 114)	M-M group (n = 38)	D-M group (n = 76)	*P*
Sufentanil								
Number of patients, n (%)	105 (19.02)	4 (10.53)	101 (19.65)	0.243	15 (13.16)	4 (10.53)	11 (14.47)	0.769
Daily average amount#, μg, mean ± SD	48.9±49.9	44.64±15.84	49.07±50.81	0.863	64.71±57.5	44.64±15.84	70.88±64.58	0.443
Exposure day##, day, mean ± SD	3.34±2.49	3.50±1.73	3.34±2.52	0.898	4.29±2.78	3.50±1.73	4.54±3.04	0.531
Fentanyl								
Number of patients, n (%)	207 (37.5)	5 (13.16)	202 (39.3)	0.002	31 (27.19)	5 (13.16)	26 (34.21)	0.031
Daily average amount #, mg, mean ± SD	0.66±0.62	0.3±0.31	0.67±0.62	0.188	0.8±1.01	0.3±0.31	0.89±1.07	0.234
Exposure day##, day, mean ± SD	3.98±2.55	2.00±1.00	4.03±2.56	0.079	3.58±2.42	2.00±1.00	3.88±2.50	0.112
Dezocine								
Number of patients, n (%)	237 (42.93)	17 (44.74)	220 (42.8)	0.950	42 (36.84)	17 (44.74)	25 (32.89)	0.303
Daily average amount#, mg, mean ± SD	14.14±10.67	15.80±12.58	14.01±10.53	0.506	15.24±10.52	15.80±12.58	14.85±9.05	0.780
Exposure day##, day, mean ± SD	4.52±2.37	4.41±2.76	4.53±2.35	0.847	4.83±2.6	4.41±2.76	5.12±2.49	0.393
Propofol								
Number of patients, n (%)	255 (46.2)	11 (28.95)	244 (47.47)	0.041	45 (39.47)	11 (28.95)	34 (44.74)	0.155
Daily average amount#, mg, mean ± SD	338.5±382.1	158.05±184.32	346.63±386.87	0.109	366.74±444.72	158.05±184.32	432.33±482.96	0.074
Exposure day##, day, mean ± SD	2.87±2.04	2.36±1.36	2.89±2.06	0.404	2.98±2.29	2.36±1.36	3.17±2.5	0.314
Probiotic								
Number of patients, n (%)	44 (7.97)	2 (5.26)	42 (8.17)	0.743	7 (6.14)	2 (5.26)	5 (6.58)	0.890
Average amount#, mg, mean ± SD	6.78±5.18	7.71±1.21	6.73±5.29	0.797	8.24±3.14	7.71±1.21	8.46±3.78	0.806
Exposure day##, day, mean ± SD	4.14±2.29	6.50±2.12	4.02±2.26	0.137	5.86±1.77	6.50±2.12	5.6±1.82	0.592
Muscle relaxants								

Number of patients, n (%)	22 (3.99)	1 (2.63)	21 (4.09)	0.990	3 (2.63)	1 (2.63)	2 (2.63)	1.000
Average amount#, mg, mean ± SD	10.75±23.17	1.14	11.20±23.64	-	6.86±6.36	1.14	9.71±5.66	-
Exposure day##, day, mean ± SD	1.95±1.68	1.00	2.00±1.7	-	1.67±1.15	1.0	2.00±1.41	-
Fat Emulsion Injection								
Number of patients, n (%)	193 (34.96)	11 (28.95)	182 (35.41)	0.529	33 (28.95)	11 (28.95)	22 (28.95)	1.000
Average amount#, ml, mean ± SD	124.09±103.5	124.68±92.71	124.06±104.35	0.983	130.61±89.04	124.68±92.71	133.57±89.22	0.795
Insulin								
Number of patients, n (%)	264 (47.83)	12 (31.58)	252 (49.03)	0.056	42 (36.84)	12 (31.58)	30 (39.47)	0.537
Average amount#, mg, mean ± SD	28.2±28.03	19.49±25.08	28.62±28.14	0.271	22.32±24.51	19.49±25.08	23.54±24.62	0.643
Kabiven								
Number of patients, n (%)	99 (17.93)	14 (36.84)	85 (16.54)	0.003	26 (22.81)	14 (36.84)	12 (15.79)	0.022
Exposure day##, day, mean ± SD	2.79±2.19	3.79±2.64	2.62±2.08	0.066	3.56±2.57	3.79±2.64	3.27±2.57	0.630
Placement of nasoenteric tubes								
Number of patients, n (%)	93 (16.85)	3 (7.89)	90 (17.51)	0.192	32 (16.84)	3 (7.89)	29 (19.08)	0.160
Average dose of metoclopramide, mg, mean ± SD	-	28.76 (8.22)	-	-		28.76 (8.22)	-	-
Average dose of domperidone, mg, mean ± SD	-	-	30.29 (5.24)	-		-	30.13 (4.37)	-
Ventilation time, days, mean ± SD	6.62 ± 1.18	7.0 ± 0.0	6.6 ± 1.21	0.328	6.7 ± 0.88	7.0 ± 0.0	6.61 ± 0.99	0.248

‡: 1 to 2 propensity score matching based on Model 1 as described in methods

#: for patients who use this drug

##: within the observational period

Figure S1: Percentage of patients who reached the 80% goal of protein or calories during observational period in the unmatched cohort (A,C) and in the 1:2 propensity score matched cohort (B, D) based on Model 1 as detailed in the methods.

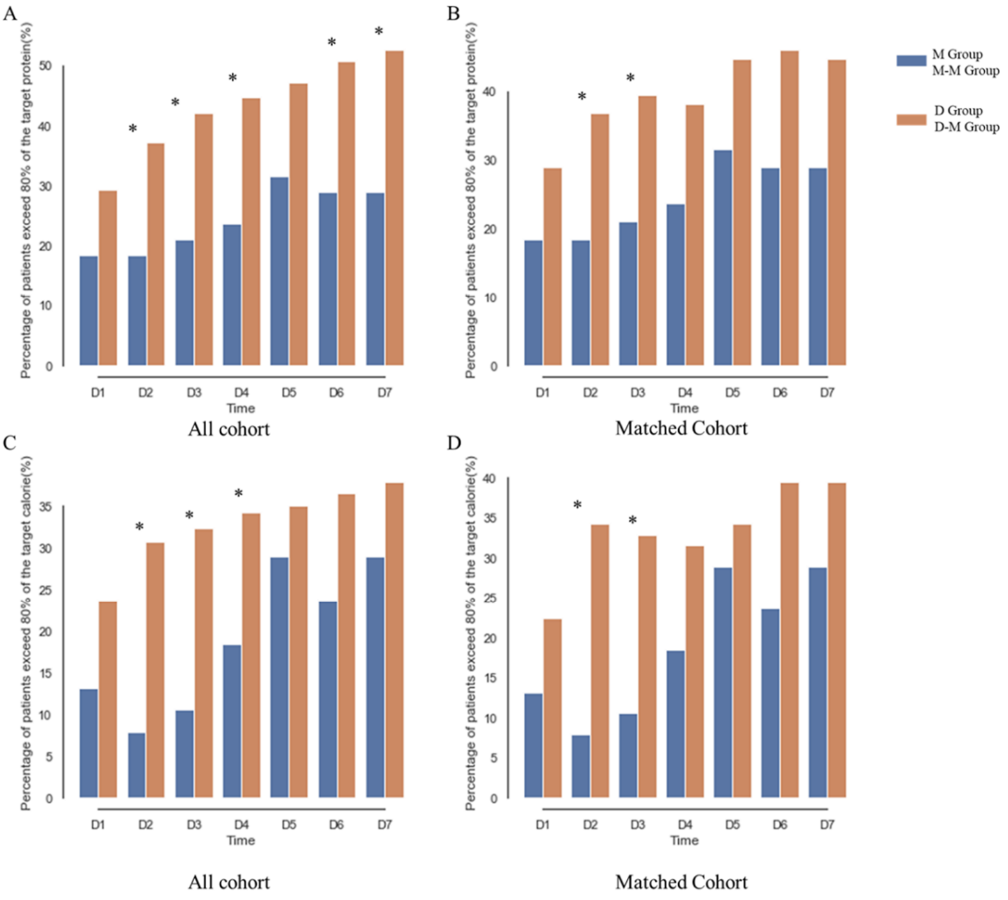

Figure S2: The amount of enteral nutrition volume, calories, and protein delivered during observational period in the unmatched cohort (A,C,E) and the 1:2 propensity score matched cohort (B, D,F) based on Model 1 as detailed in the methods.

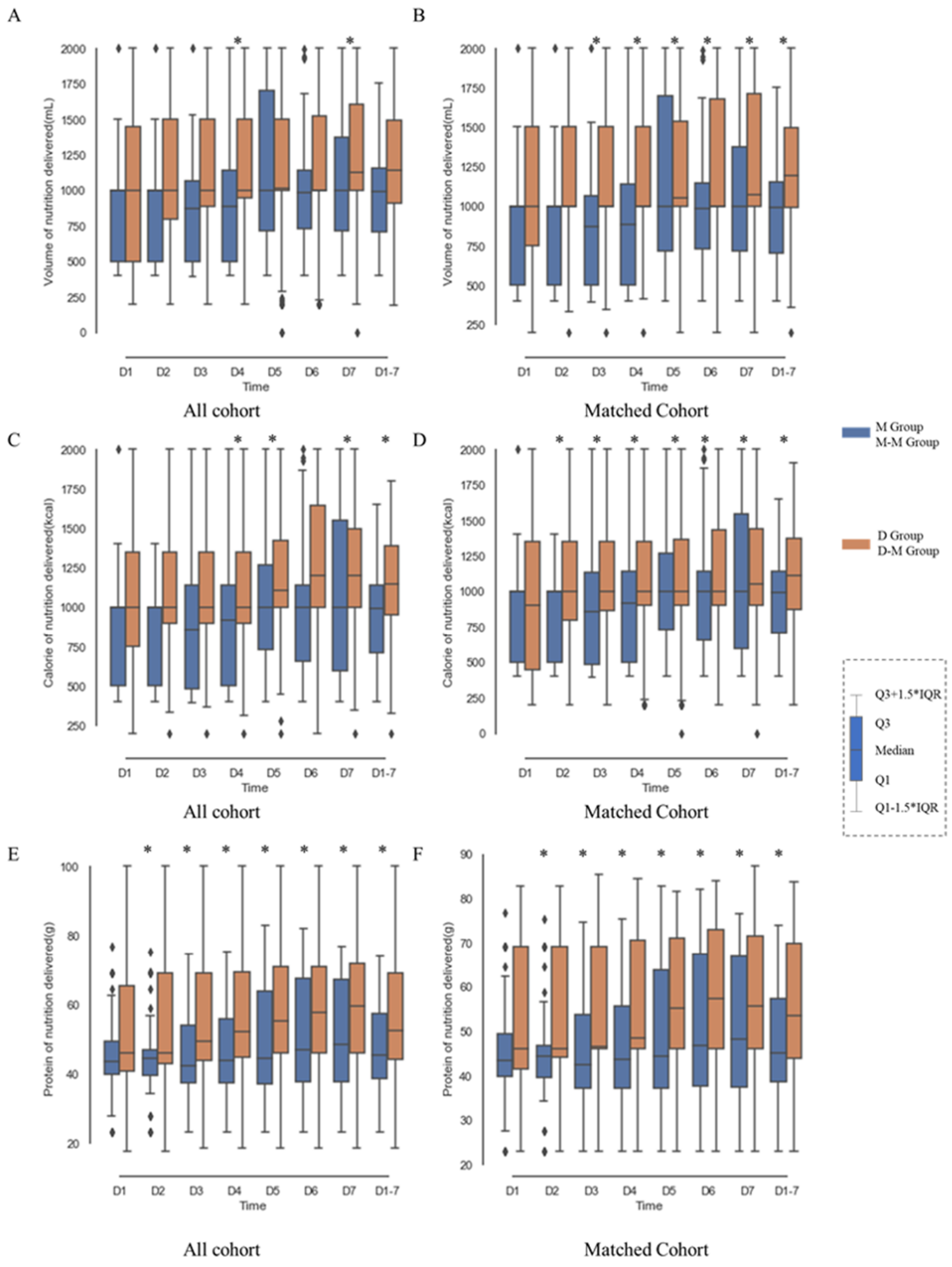

Table S5 Univariate regression model for primary outcome in unmatched and matched cohorts

Variable	Unmatched cohort				Matched cohort[†]			
	Feeding success[††], # (%)		Odds Ratio (95% CI)	*P*	Feeding success[††], # (%)		Odds Ratio (95% CI)	*P*
	No	Yes			No	Yes		
EFI treatment								
Metoclopramide	30 (78.95)	8 (21.05)	1 [Reference]	0.014	30 (78.95)	8 (21.05)	1 [Reference]	0.040
Domperidone	298 (57.98)	216 (42.02)	2.718 (1.222-6.405)		45 (59.21)	31 (40.89)	2.583 (1.046-6.380)	
Feeding start time[∫]								
Within 7 day	294 (57.98)	213 (42.02)	1 [Reference]	0.024	69 (64.49)	38 (35.51)	1 [Reference]	0.277
≥ 7 day	34 (75.56)	11 (24.44)	0.447 (0.221-0.901)		6 (85.71)	1 (14.29)	0.303 (0.035-2.608)	
Supplementary Parenteral nutrition[∫∫]								
No	176 (57.70)	129 (42.30)	1 [Reference]	0.248	40 (61.54)	25 (38.46)	1 [Reference]	0.272
Yes	152 (61.54)	95 (38.46)	0.853 (0.605-1.201)		35 (71.42)	14 (28.57)	0.640 (0.289-1.419)	
Insulin[∫∫]								
No	175 (60.76)	113 (39.24)	1 [Reference]	0.502	49 (68.06)	23 (31.94)	1 [Reference]	0.505
Yes	153 (57.95)	111 (42.04)	1.124 (0.800-1.579)		26 (61.90)	16 (38.10)	1.311 (0.592-2.905)	
Probiotic[∫∫]								
No	300 (59.05)	208 (40.94)	1 [Reference]	0.553	70 (65.42)	37 (34.58)	1 [Reference]	0.746
Yes	28 (63.63)	16 (36.37)	0.824 (0.435-1.562)		5 (71.43)	2 (28.57)	0.757 (0.140-4.091)	
Opioid[∫∫]								
No	99 (68.27)	46 (31.72)	1 [Reference]	0.012	32 (76.19)	10 (23.81)	1 [Reference]	0.077
Yes	229 (56.26)	178 (43.73)	1.637 (1.120-2.498)		43 (59.72)	29 (40.28)	2.158 (0.921-5.060)	
CRRT[∫∫]								
No	303 (58.83)	212 (41.17)	1 [Reference]	0.298	71 (65.74)	37 (34.26)	1 [Reference]	0.963
Yes	25 (67.56)	12 (32.43)	0.686 (0.337-1.396)		5 (71.43)	2 (28.57)	0.959 (0.168-5.484)	

Propofol∬								
No	185 (62.29)	112 (37.71)	1 [Reference]	0.139	49 (71.01)	20 (28.99)	1 [Reference]	0.147
Yes	143 (56.08)	112 (43.92)	1.294 (0.920-1.819)		26 (57.78)	19 (41.30)	1.790 (0.814-3.936)	
Muscle relaxant∬								
No	317 (59.81)	213 (40.19)	1 [Reference]	0.361	73 (65.77)	38 (34.23)	1 [Reference]	0.974
Yes	11 (50.00)	11 (50.00)	1.488 (0.634-3.495)		2 (66.67)	1 (33.33)	0.961 (0.084-10.935)	
Placement of nasoenteric tube∬								
No	264 (57.52)	195 (42.48)	1 [Reference]	0.044	64 (65.31)	34 (34.69)	1 [Reference]	0.788
Yes	64 (68.82)	29 (31.18)	0.613 (0.381-0.988)		11 (68.75)	5 (31.25)	0.856 (0.275-2.665)	
Vasopressor∬								
No	220 (58.20)	158 (41.79)	1 [Reference]	0.390	56 (67.47)	27 (32.53)	1 [Reference]	0.537
Yes	108 (62.07)	66 (37.93)	0.851 (0.589-1.230)		19 (61.29)	12 (38.71)	1.310 (0.556-3.084)	
SOFA score$^{\Delta}$								
< 4	141 (58.75)	99 (41.25)	1 [Reference]	0.779	46 (63.01)	27 (36.99)	1 [Reference]	0.405
≥ 4	187 (59.94)	125 (40.06)	0.952 (0.676-1.341)		29 (70.73)	12 (29.27)	0.705 (0.309-1.606)	
NUTRIC score$^{\Delta}$								
< 4	99 (57.89)	72 (42.11)	1 [Reference]	0.625	22 (56.41)	17 (43.59)	1 [Reference]	0.130
≥ 4	229 (60.10)	152 (39.90)	0.913 (0.633-1.316)		53 (70.67)	22 (29.33)	0.537 (0.240-1.201)	
Age								
< 54	176 (60.69)	114 (39.31)	1 [Reference]	0.523	35 (67.31)	17 (32.69)	1 [Reference]	0.754
≥ 54	152 (58.02)	110 (41.98)	1.117 (0.795-1.570)		40 (64.52)	22 (35.48)	1.132 (0.520-2.467)	
Gender								
Female	98 (53.55)	85 (46.45)	1 [Reference]	0.048	23 (63.89)	13 (36.11)	1 [Reference]	0.771
Male	230 (62.33)	139 (37.67)	0.679 (0.487-0.998)		52 (66.67)	26 (33.33)	0.885 (0.387-2.023)	
Admission type								
Post elective surgery	117 (58.79)	82 (41.21)	1 [Reference]		26 (66.67)	13 (33.33)	1 [Reference]	

Post emergency surgery	31 (64.58)	17 (31.42)	0.782 (0.406-1.507)	0.463	6 (66.67)	3 (33.33)	1.000 (0.215-4.653)	1.000
From ward	63 (64.95)	34 (35.05)	0.770 (0.465-1.274)	0.309	15 (68.18)	7 (31.82)	0.933 (0.305-2.853)	0.904
From emergency department	110 (56.70)	84 (43.30)	1.090 (0.730-1.626)	0.675	25 (60.98)	16 (39.02)	1.280 (0.513-3.195)	0.597
Transfer from other hospital	7 (50.00)	7 (50.00)	1.427 (0.482-4.223)	0.521	3 (100.00)	0 (0.00)	-	-
APACHE score$^{\Delta}$								
< 18	128 (56.89)	97 (43.11)	1 [Reference]	0.315	27 (61.36)	17 (38.64)	1 [Reference]	0.430
≥ 18	200 (61.16)	127 (38.84)	0.838 (0.593-1.183)		48 (68.57)	22 (31.43)	0.728 (0.331-1.603)	
Surgical$^{\Delta\Delta}$								
No	168 (59.36)	115 (40.64)	1 [Reference]	0.978	42 (67.74)	20 (32.26)	1 [Reference]	0.632
Yes	160 (59.48)	109 (40.52)	0.995 (0.708-1.398)		33 (63.46)	19 (36.54)	1.209 (0.556-2.627)	
Hospitalization time before ICU admission								
< 4 days	251 (59.20)	173 (40.80)	1 [Reference]	0.847	55 (67.07)	27 (32.93)	1 [Reference]	0.644
≥ 4 days	77 (60.16)	51 (39.84)	0.961 (0.642-1.438)		20 (62.50)	12 (37.50)	1.222 (0.522-2.863)	
BMI								
< 23	80 (53.33)	70 (46.67)	1 [Reference]	0.333	15 (65.22)	8 (34.78)	1 [Reference]	0.948
≥ 23	238 (60.71)	154 (39.29)	0.832 (0.573-1.207)		60 (65.93)	31 (34.07)	0.969 (0.370-2.534)	

†: 1 to 2 propensity score matching based on model 1 as described in methods

††: Average DPP % > 80%

∫: calculated from ICU admission

∬: within the observational period

$^{\Delta}$: at ICU admission

$^{\Delta\Delta}$: Post-surgery or experienced major surgery within 48 hours prior to ICU admission

EFI: enteral feeding intolerance; BMI: Body mass index

Table S6 Primary outcomes by propensity score matching using different ratios based on Model 1

Variables	1:1		1:3		1:4		1:5	
	Feeding Success‡	*P* value	Feeding Success‡	*P* value	Feeding Success‡	*P* value	Feeding Success‡	*P* value
All	24 (31.58)		61 (40.13)		81 (42.63)		96 (42.11)	
M-M group	8 (21.05)	0.048	8 (21.05)	0.010	8 (21.05)	0.005	8 (21.05)	0.007
D-M group	16 (42.11)		53 (46.49)		73 (48.03)		88 (46.32)	

‡: defined as average DPP % > 80%

Figure S3. Percentage of daily protein prescription in protein goal (DPP %) through the observational period in (A) 1:1, (B) 1:3, (C) 1:4, and (D) 1:5 propensity score matched cohorts based on Model 1 as detailed in the methods.

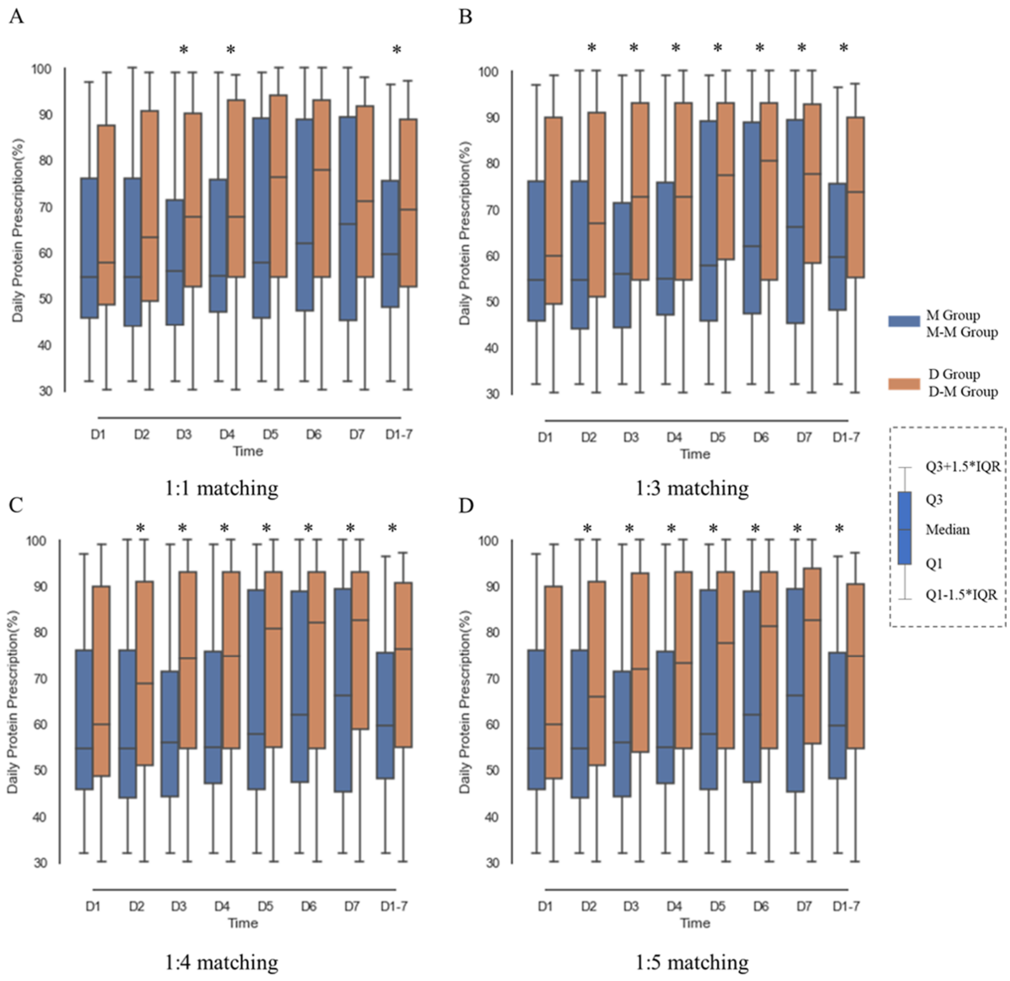

Table S7 Primary outcomes by propensity score matching using different ratios based on Model 2

Variables	1:1		1:2		1:3		1:4	
	Feeding Success†	*P* value	Feeding Success†	*P* value	Feeding Success†	*P* value	Feeding Success†	*P* value
All	29 (38.16)		41 (35.96)		55 (36.18)		71 (37.37)	
M-M group	8 (21.05)	0.005	8 (21.05)	0.032	8 (21.05)	0.041	8 (21.05)	0.033
D-M group	21 (55.26)		33 (43.42)		47 (41.23)		63 (41.45)	

†: defined as average DPP % > 80%

Figure S4. Percentage of daily protein prescription in protein goal (DPP%) through the observational period in (A) 1:1, (B) 1:2, (C) 1:3, and (D) 1:4 propensity score matched cohorts based on Model 2 as detailed in the methods.

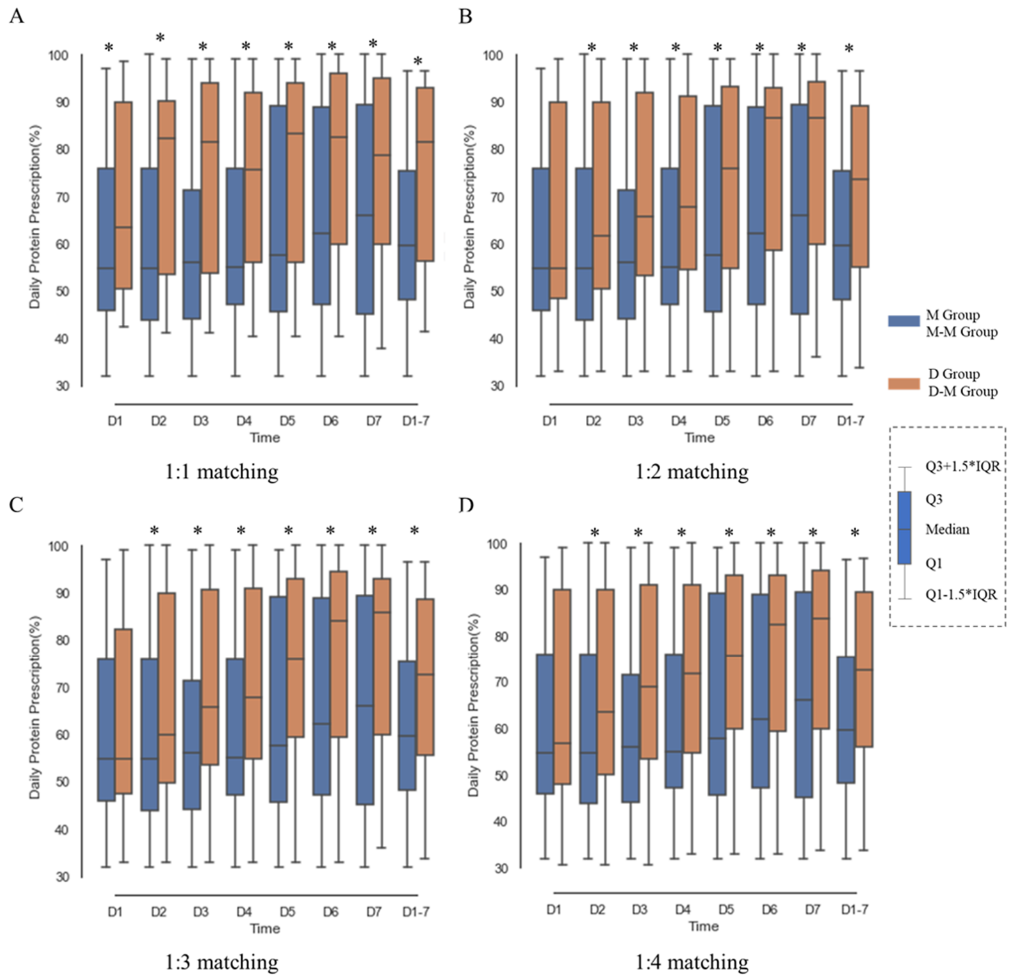

Supplement: Supplementary file 1 [file jpm-11-00846-s001.zip › jpm-1337893-supplementary.pdf]
